# Defining the molecular profile of planarian pluripotent stem cells using a combinatorial RNA-seq, RNA interference and irradiation approach

**DOI:** 10.1186/gb-2012-13-3-r19

**Published:** 2012-03-22

**Authors:** Jordi Solana, Damian Kao, Yuliana Mihaylova, Farah Jaber-Hijazi, Sunir Malla, Ray Wilson, Aziz Aboobaker

**Affiliations:** 1 Centre for Genetics and Genomics, University of Nottingham, Queen's Medical Centre, Nottingham NG7 2UH, UK; 2 Deep Seq, Faculty of Medical and Health Sciences, University of Nottingham, Queen's Medical Centre, Nottingham NG7 2UH, UK

## Abstract

**Background:**

Planarian stem cells, or neoblasts, drive the almost unlimited regeneration capacities of freshwater planarians. Neoblasts are traditionally described by their morphological features and by the fact that they are the only proliferative cell type in asexual planarians. Therefore, they can be specifically eliminated by irradiation. Irradiation, however, is likely to induce transcriptome-wide changes in gene expression that are not associated with neoblast ablation. This has affected the accurate description of their specific transcriptomic profile.

**Results:**

We introduce the use of *Smed-histone-2B *RNA interference (RNAi) for genetic ablation of neoblast cells in *Schmidtea mediterranea *as an alternative to irradiation. We characterize the rapid, neoblast-specific phenotype induced by *Smed-histone-2B *RNAi, resulting in neoblast ablation. We compare and triangulate RNA-seq data after using both irradiation and *Smed-histone-2B *RNAi over a time course as means of neoblast ablation. Our analyses show that *Smed-histone-2B *RNAi eliminates neoblast gene expression with high specificity and discrimination from gene expression in other cellular compartments. We compile a high confidence list of genes downregulated by both irradiation and *Smed-histone-2B *RNAi and validate their expression in neoblast cells. Lastly, we analyze the overall expression profile of neoblast cells.

**Conclusions:**

Our list of neoblast genes parallels their morphological features and is highly enriched for nuclear components, chromatin remodeling factors, RNA splicing factors, RNA granule components and the machinery of cell division. Our data reveal that the regulation of planarian stem cells relies on posttranscriptional regulatory mechanisms and suggest that planarians are an ideal model for this understudied aspect of stem cell biology.

## Background

Understanding stem cells has become a major goal of molecular biology. Several studies have started to decipher the mechanisms that regulate pluripotency in the stem cells of the mammalian inner cell mass, or their *in vitro *counterparts [1-3]. However, model systems with appropriate life histories, such as freshwater planarians, offer a valuable comparative platform to understand the evolutionary basis of animal pluripotency and self-renewal. Planarian neoblasts (NBs) are pluripotent stem cells that drive the almost unlimited regenerative power of freshwater planarians [4,5]. Furthermore, NBs also drive the homeostatic cell turnover of intact worms. NBs are distributed in large numbers throughout the planarian parenchyma and therefore make planarians an amenable model system for regeneration and stem cell biology. Furthermore, a series of molecular techniques have been developed for planarians and this, together with their amenability to RNA interference (RNAi) [6] and the availability of genomic and transcriptomic sequences [7-12], has yielded a panoply of studies describing genes expressed in NBs and their functions.

NBs have classical stem cell morphology [13,14]. They are small cells, ranging from 6 to 12 μm in diameter, and consist of a large nucleus and very little cytoplasm. They were classically considered undifferentiated cells because of their open chromatin when visualized under electron microscopy. The most characteristic feature of NB cells is the presence of chromatoid bodies (CBs) in their cytoplasm [13]. CBs are electron dense granules located in the perinuclear region, often associated with nuclear pores and mitochondria. These structures have both morphological and molecular similarities to germ granules present in the germ line cells of metazoans (collectively known as 'nuage'). A wide variety of known germ cell markers are also present in NBs. Significantly, some are required for NB function and are necessary for both regeneration and the homeostatic cell turnover of the worms. These include two Piwi homologues [15,16] and a Bruno [17], a Pumilio [18] and a Tudor homologue gene [19], all involved in post-transcriptional regulation. Abrogation of these genes by RNAi leads to failures in regeneration and cell turnover after gene knockdown. Some of these markers are also expressed in the central nervous system (CNS) of planarians [15,17,18,20-22], suggesting a shared requirement for complex posttranscriptional/RNA-based gene regulation in NBs and neurons.

One accepted definition of planarian stem cells is the fact that they are the only proliferative cell type in asexual races of planarians. This observation, from the classical literature, has been confirmed by bromodoxyuridine incorporation studies and phospho-histone-3 labeling of mitotic cells [23]. This distinctive feature of NBs has made irradiation the method of choice to ablate NBs from planarians. Irradiation eliminates all NBs in a period of 24 to 48 hours, and the cells described as their recent post-mitotic progeny also subsequently disappear as they differentiate and are not replaced [24]. This has allowed expression profiling of animals with and without NBs. Not surprisingly, however, this approach is limited by the non-specific effects introduced by whole organism irradiation. Several studies have attempted the description of NB transcriptomic [24,25] and proteomic profiles [26], but the non-specific effects of irradiation are likely to introduce false positives in these kinds of studies.

Here, we introduce the use of *Smed-histone-2B *(*Smed-H2B*) RNAi for genetic ablation of NBs as an alternative and more specific method than irradiation. *Smed-H2B *was first described by Guo and co-workers [17] as a NB-specific histone variant that could be used as a marker for NBs, and had a potent and specific phenotype. We describe and characterize in detail its phenotype, which eliminates all NB marker expression in a period of only 5 days after the injection. We use it to eliminate NB-specific gene expression and obtain transcriptomic data by RNA-seq. We compare these data to a parallel study using irradiation and demonstrate that *Smed-H2B *RNAi has increased specificity with regards to identifying transcripts expressed in NBs. We combine data from both ablation methods to identify a high confidence set of genes expressed in NBs. We validate these data *in vivo *and finally describe the molecular pathways that are present in planarian stem cells using Gene Ontology (GO) and Kyoto Encyclopedia of Genes and Genomes (KEGG) annotations. Our studies uncover that NBs are highly enriched for nuclear components, chromatin proteins, RNA binding proteins and other germ granule components and cell division machinery. Also, they confirm a functional link between NBs and neurons, probably due to the presence of neuronal RNA granules in neuron cells [27]. Finally, we observe a very strong enrichment of factors related to RNA splicing and RNA transport, uncovering that the regulation of NB stem cell biology relies heavily on posttranscriptional gene regulation (PTGR).

## Results

### *Smed-H2B *is expressed in NBs but not in the CNS

**Figure 1:**
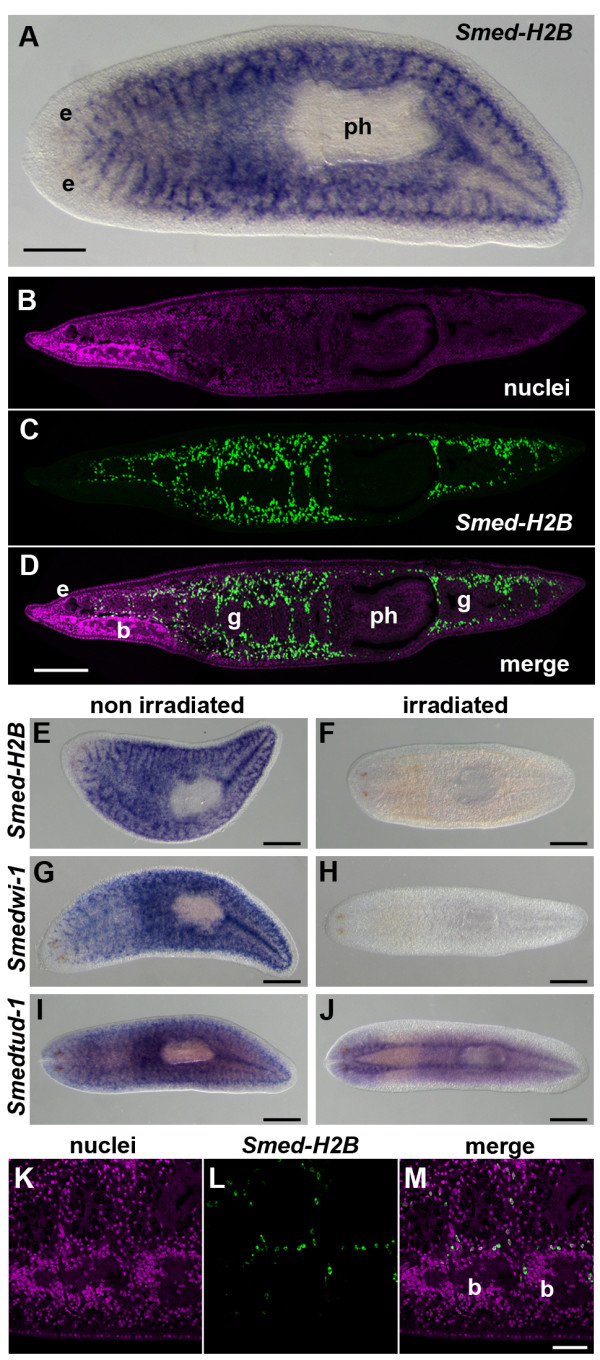
***Smed-H2B *is expressed in NBs but not in the CNS**. **(a) **WMISH of *Smed-H2B*, showing the characteristic expression pattern of genes expressed in NBs. **(b-d) **Sagital section of a FISH experiment on histological sections of *Smed-H2B *(c), counterstained with nuclei staining (b), and a merged image (d). *Smed-H2B*-positive cells are distributed in the parenchyma of the animal, absent from the pharynx (ph), the most anterior tip of the head, gut (g), and brain (b), consistent with its expression in NBs. **(e-j) **WMISH of *Smed-H2B *(e,f), *Smedwi-1 *(g,h) and *Smedtud-1 *(i,j) in non-irradiated (e,g,i) and irradiated specimens (f,h,j). All the *Smed-H2B *signals disappear after irradiation, similar to *Smedwi-1 *and unlike *Smedtud-1*, which retains prominent expression in the planarian CNS. **(k-m) **Detail of a sagital section of a FISH experiment on histological sections of *Smed-H2B *(l), counterstained with nuclei staining (k), and a merged image (m). No expression of *Smed-H2B *is detected in brain cells. b, brain, g, gut; e, eye; ph, pharynx. Anterior is to the left in all panels. Scale bars: 500 μm (a-j); 50 μm (k-m).

We first characterized the expression pattern and phenotype of *Smed-H2B*, a Histone H2B variant known to be expressed in NBs [17]. Whole mount *in situ *hybridization (WMISH) experiments revealed a NB-like expression pattern (Figure 1a), similar to the one previously described [17]. Fluorescent *in situ *hybridization (FISH) on paraffin sections detected *Smed-H2B*-positive cells throughout the parenchyma, in a pattern similar to that of NBs (Figure 1b-d). No cells were detected in the pharynx and in the most anterior tip of the head. Expression of *Smed-H2B *was also irradiation sensitive (Figure 1e-j), with all expression localized to irradiation-sensitive cells. We also analyzed the expression of *Smedwi-1*, a gene specifically expressed in NBs [16], and staining was lost similarly after irradiation (Figure 1g,h). We also analyzed the expression of *Smedtud-1*, whose homologue in the planarian species *Schmidtea polychroa *was described to be expressed in the brain at both the mRNA and protein levels. A significant part of *Schmidtea mediterranea*'s expression does not correspond to NBs and localizes to the CNS (Figure 1i,j). *Smed-H2B-*positive cells were detected near the brain and sometimes within the borders of the brain structure by FISH (Figure 1k-m). No expression was detected in brain cells themselves, and the positive cells observed inside the structure of the brain likely correspond to NBs that are infiltrating the brain as a part of the homeostatic tissue turnover of the animal. These results show that *Smed-H2B *is expressed specifically in NBs and is not detected in any other tissue of the planarian body, including the CNS.

### *Smed-H2B *RNAi induces very rapid and specific NB loss

**Figure 2:**
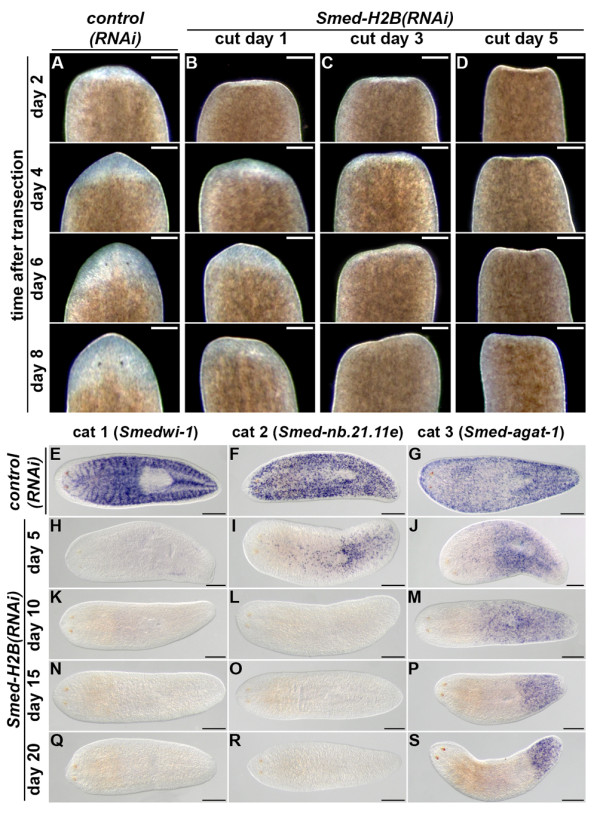
***Smed-H2B *RNAi induces a very quick and specific NB loss**. **(a-d) ***Control(RNAi) *(a) and *Smed-H2B(RNAi) *animals cut 1 (b), 3 (c) and 5 (d) days after RNAi, and monitored every 2 days after transection. Only anterior wounds are shown. Only one *control(RNAi) *time point (5 days) is shown as no differences were detected among them. All *Smed-H2B(RNAi) *animals fail to regenerate missing tissues, but the earlier time points (b,c) are able to generate a small blastema (white tissue). **(e-s) **WMISH of *Smedwi1 *(e,h,k,n,q), *Smed-nb.21.11e *(f,i,l,o,r) and *Smed-agat-1 *(g,j,m,p,s) in *control(RNAi) *(e-g) and *Smed-H2B(RNAi) *animals 5 (h-j), 10 (k-m), 15 (n-p) and 20 (q-s) days after RNAi. Only one *control(RNAi) *time point (20 days) is shown since no differences were detected among them. *Smedwi-1*-positive cells disappear quickly after *Smed-H2B(RNAi) *(h,k,n,q). *Smed-nb.21.11e*-positive cells are still detectable 5 days after *Smed-H2B(RNAi) *(i) but disappear soon after (l,o,r). *Smed-agat-1-*positive cells progressively disappear and are restricted to the posterior part of the animal (j,m,p,s). (a-d) Anterior is to the top. (e-s) Anterior is to the left. Scale bars: 500 μm. The lables cat 1, cat 2 and cat 3 refer to the definitions previously used by Esienhoffer et al [24].

We performed RNAi knockdown experiments in order to study the effect of *Smed-H2B *knockdown in NBs. All animals after *Smed-H2B *RNAi were unable to regenerate. Furthermore, this phenotype was observed very shortly after the delivery of double-stranded RNA (dsRNA). Animals cut one day after RNAi were able to produce a small regeneration blastema that never progressed to complete regeneration (Figure 2b versus Figure 2a). Animals cut after 3 days produced an even smaller blastema (Figure 2c) and animals cut after 5 days of RNAi never produced a visible regeneration blastema (Figure 2d).

We analyzed the dynamics of NBs and their progeny after *Smed-H2B *RNAi (Figure 2e-s). *Smed-H2B(RNAi) *animals showed a dramatic and unrecoverable decrease in NB number only 5 days after dsRNA delivery (Figure 2h,k,n,q versus Figure 2E), as detected by the use of the NB-specific probe *Smedwi-1*. We also analyzed the expression patterns of the NB progeny-specific genes *Smed-nb.21.11e *and *Smed-agat-1 *[24]. Only 5 days after dsRNA delivery, *Smed-nb.21.11e*-positive cells were dramatically reduced in numbers (Figure 2i versus Figure 2f), and became undetectable 10 days after dsRNA administration (Figure 2l) and at later time points (Figure 2o,r). This *Smed-nb.21.11e*-positive cell loss resembles the disappearance of this marker upon irradiation [24], but at a reduced speed (Additional file 1). We also analyzed the expression of *Smed-agat-1*, a marker of later NB progeny [24]. Similar to the dynamics after irradiation, although slower (Additional file 2), 5 days after RNAi *Smed-agat-1*-positive cells were greatly reduced at the anterior region of the organisms (Figure 2j versus Figure 2g), and progressively disappeared at later time points (Figure 2m,p,s), although a complete disappearance was not observed 20 days after RNAi (Figure 2s). These results show that *Smed-H2B *RNAi rapidly removes NBs and is unparalleled by any other described RNAi phenotypes [16-19,21].

### *Smed-H2B *RNAi does not affect differentiated cell types and tissues

**Figure 3:**
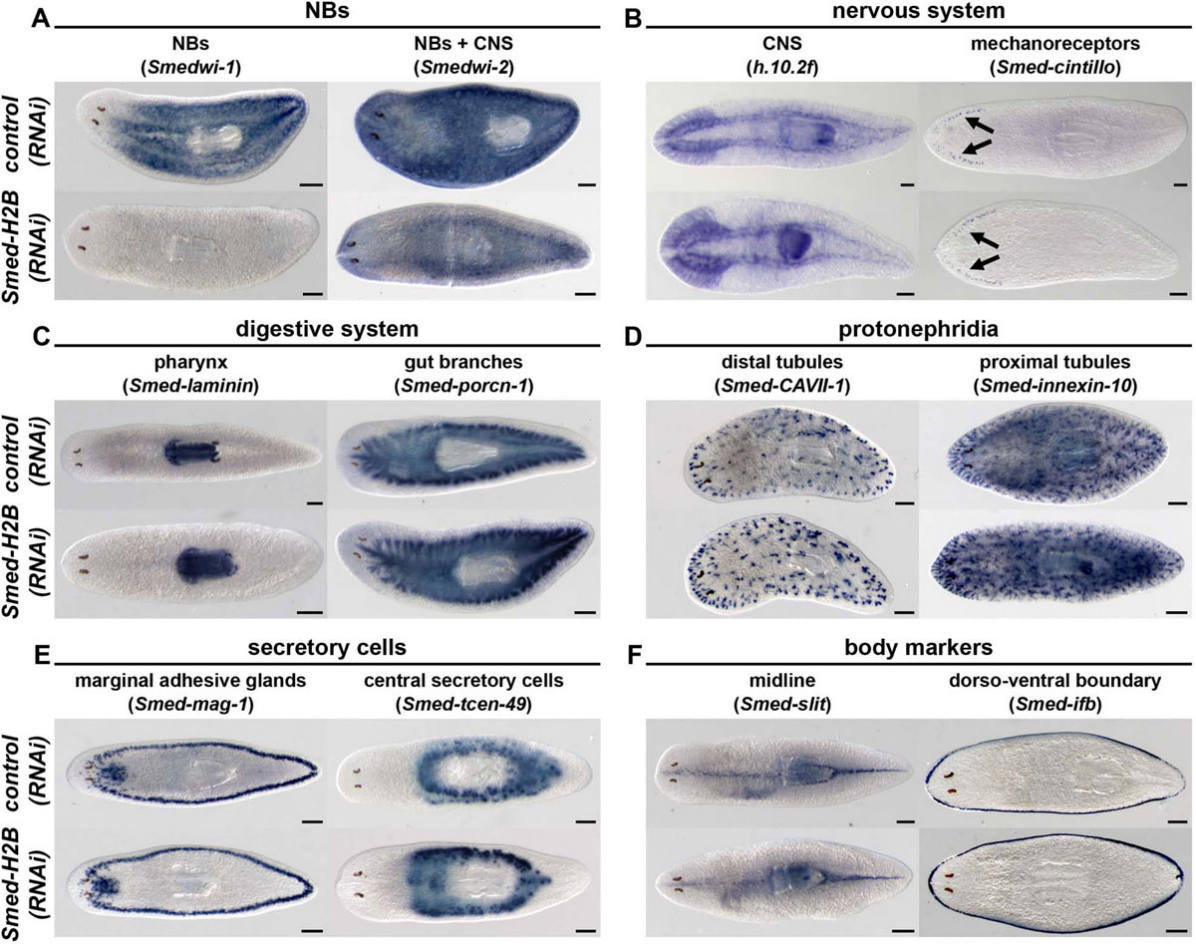
***Smed-H2B *RNAi does not affect differentiated cell types and tissues**. **(a-f) **WMISH of the neoblast markers *Smedwi-1 *and *Smedwi-2 *(also expressed in the CNS) (a), the nervous system markers *h.10.2f *and *Smed-cintillo *(arrows) (b), the digestive system markers *Smed-laminin *and *Smed-porcn-1 *(c), the protonephridial markers *Smed-CAVII-1 *and *Smed-innexin-10 *(d), the secretory cell markers *Smed-mag-1 *and *Smed-tcen49 *(e) and the body positional markers *Smed-slit *and *Smed-ifb *(f) in *control(RNAi) *(top panels) and *Smed-H2B(RNAi) *(bottom panels) animals 5 days after RNAi. *Smed-H2B(RNAi) *animals show a dramatic decrease of *Smedwi-1 *and *Smedwi-2 *signals 5 days after RNAi (a), but no significant difference for any differentiated cell marker (b-f). Anterior is to the left. Scale bars: 500 μm.

We then analyzed if *Smed-H2B(RNAi) *animals had normal expression patterns of differentiated cell type markers 5 days after RNAi, a time point at which NBs were depleted (Figure 3a). We checked the expression pattern of the nervous tissue markers *h.10.2f *[28] and *Smed-cintillo *[29] (Figure 3b), the pharynx and gut markers *Smed-laminin *[30] and *Smed-porcn-1 *[31] (Figure 3c), the protonephridial cell markers *Smed-CAVII-1 *and *Smed-inx10 *[32] (Figure 3d), and the secretory cell type markers *Smed-mag1 *[33] and *Smed-tcen49 *[34,35] (Figure 3e). No differences were observed for any of these markers. Furthermore, *Smed-H2B(RNAi) *animals did not show any morphologic defect at early time points - for example, the midline marker *Smed-slit *[36] and the dorso-ventral margin marker *Smed-ifb *[37,38]. Taken together, these results show that while *Smed-H2B *RNAi specifically and rapidly affects NBs, there are no early effects on the maintenance of differentiated cells.

### Early dynamics of NB loss upon *Smed-H2B *RNAi

**Figure 4:**
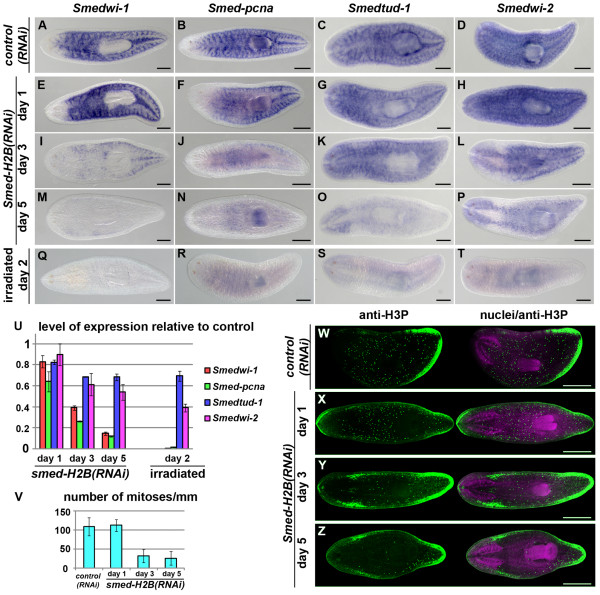
**Early dynamics of NB loss on *Smed-H2B(RNAi) *animals**. **(a-t) **WMISH of *Smedwi1 *(a,e,i,m,q), *Smed-pcna *(b,f,j,n,r), *Smedtud-1 *(c,g,k,o,s) and *Smedwi-2 *(d,h,l,p,t) in *control(RNAi) *(a-d) and *Smed-H2B(RNAi) *animals 1 (e-h), 3 (i-l) and 5 (m-p) days after RNAi, and irradiated animals (q-t). Only one *control(RNAi) *time point (20 days) is shown since no differences were detected among them or non-irradiated controls. Most signals located in NBs disappeared progressively for all markers (e-p). Almost no signals were detected 5 days after RNAi for the NB-specific markers *Smedwi-1 *and *Smed-pcna *(m,n), similar to irradiated animals (q,r). The expression in the CNS of *Smedtud-1 *(o) and *Smedwi-2 *(p) was not eliminated by *Smed-H2B *RNAi, similar to irradiated animals (s,t). **(u) **Quantification of the level of expression by quantitative RT-PCR of Smed*wi-1*, *Smed-pcna*, *Smedtud-1 *and Smed*wi-2 *in *Smed-H2B(RNAi) *animals 1, 3 and 5 days after RNAi (left), normalized expression and relative to *control(RNAi) *samples and in irradiated animals (right), normalized expression and relative to non-irradiated animals. Error bars represent standard deviation. The expression level of all markers is downregulated after NB ablation by *Smed-H2B *RNAi or irradiation, but a considerable portion of the expression of *Smedtud-1 *and *Smedwi-2 *is still detected after NB ablation. **(v) **Quantification of mitosis by counting of phospho-histone-3 (H3P)-positive cells in WMIHC on *control(RNAi) *and *Smed-H2B(RNAi) *animals 1, 3 and 5 days after RNAi (N = 5 per time point). *Smed-H2B(RNAi) *animals have significantly reduced numbers of mitotic cells 5 days after RNAi. **(w-z) **WMIHC with an anti-H3P counterstained with nuclear staining (nuclei/anti-H3P) in *control(RNAi) *(w) and *Smed-H2B(RNAi) *animals 1 (x), 3 (y) and 5 (z) days after RNAi. The mitosis of *Smed-H2B(RNAi) *animals tended to disappear progressively (z). (a-t,w-z) Anterior is to the left. Scale bars: 500 μm.

In order to further assess *Smed-H2B *RNAi as a tool for NB ablation, we looked at several known NB markers in *control(RNAi) *(Figure 4a-d) and *Smed-H2B(RNAi) *animals at one (Figure 4e-h), three (Figure 4I-L) and five days (Figure 4M-P) after dsRNA delivery and compared these to irradiation (Figure 4q-t). We selected *Smedwi-1 *and *Smed-pcna *as candidate genes for expression exclusively in NBs [16,39] and *Smedtud-1 *and *Smedwi-2 *[15,16,19] as genes expressed in NBs and the CNS. No clear effect on the expression pattern of these four genes was detected one day after *Smed-H2B *RNAi (Figure 4e-h versus Figure 4a-d). Three days after *Smed-H2B *RNAi, however, the staining of all four genes was dramatically reduced (Figure 3i-l) and 5 days after the third injection, and consistent with our previous experiments, the NB-specific staining of all four genes disappeared almost completely (Figure 4m-p). Similar to irradiation (Figure 4q-t), no staining was observed for *Smedwi-1 *and *Smed-pcna *while the staining corresponding to the CNS expression is still observed for *Smedtud-1 *and *Smedwi-2*. In addition, the expression of *Smed-mcm2 *and *Smedwi-3 *[15,40] followed the same dynamics after *Smed-H2B *RNAi (Additional file 3). Expression of these genes was also observed in two clusters of dorsal cells, particularly visible for *Smedwi-2 *(Figure 4p), *Smed-mcm2 *and *Smedwi-3 *(Additional file 3). These clusters likely correspond to *Smed-nanos*-positive cells, which are believed to be NB-like germ cell precursors [41-43]. Consistently, when we analyzed the dynamics of *Smed-nanos *after *Smed-H2B *RNAi, we observed that these *Smed-nanos*-positive cells were, although severely reduced in numbers, still present 5 days after dsRNA delivery (Additional file 4), probably reflecting a slower turnover of these germ cell precursors found in asexual worms.

### The CNS component of expression of some NB markers persists after NB ablation

We quantified the disappearance of the same four transcripts by quantitative RT-PCR (qRT-PCR), and compared the levels to those of *control(RNAi) *animals, non-irradiated wild-type animals and irradiated wild-type animals (Figure 4u). We observed that *Smedwi-1 *and *Smed-pcna *transcripts progressively disappeared over 5 days, with around 10% of the expression of controls observed after 5 days. Expression of these two mRNAs after irradiation was almost undetectable. This difference between *Smed-H2B(RNAi) *animals and irradiated animals can be explained by the presence of *Smed-nanos*-positive cells in *Smed-H2B(RNAi) *animals after 5 days of RNAi. The expression levels of the two genes expressed in NBs and the CNS decreased less dramatically. *Smedtud-1 *and *Smedwi-2 *transcripts go down to about 70% and 50% the level of control animals, respectively. After irradiation, expression was found to be reduced as well, with about 70% of the *Smedtud-1 *and 40% of the *Smedwi-2 *mRNA expression remaining. The levels observed after 5 days of *Smed-H2B *RNAi and irradiation are similar and likely represent expression in irradiation-insensitive cells within the CNS (as indicated by WMISH). Taken together, these results show that *Smed-H2B *RNAi induces a decrease in the mRNA levels of several NB markers that parallels the decrease induced by irradiation and therefore likely induces a dramatic and relatively rapid loss of NBs. Significant proportions of transcripts that are also expressed in the CNS are still detected by *in situ *hybridization and qRT-PCR after NB ablation by either irradiation or *Smed-H2B *RNAi.

### *Smed-H2B *RNAi eliminates mitotic activity

In order to test if NBs were still able to proliferate, we performed whole mount immunohistochemistry (WMIHC) of the mitotic marker phospho-histone-3 and counted the number of cells that were undergoing mitosis. Consistent with the results observed for the *in situ *stainings of NB markers, we observed that the mitotic numbers were not altered one day after RNAi (Figure 4v), but quickly decreased soon after that. By day 3 and by day 5 mitotic numbers were severely decreased. Interestingly, similar to what is observed by *in situ *hybridization, the few remaining mitotic cells observed after 3 and 5 days of the third injection accumulated in the posterior part of the animal (Figure 4y,z versus Figure 4w,x). This pattern of NB loss has been observed after different RNAi treatments [19,44].

### *Smed-H2B *RNAi induces a peak of progeny cells

**Figure 5:**
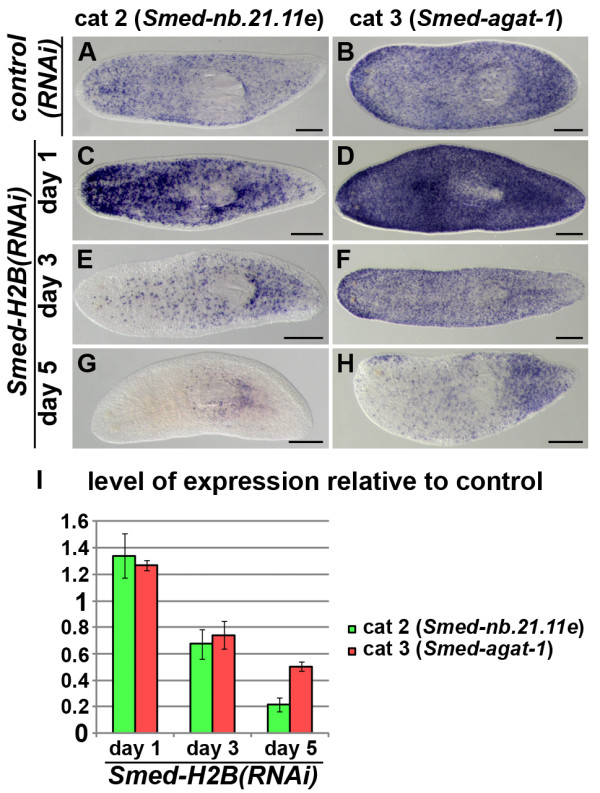
***Smed-H2B *RNAi induces a peak of progeny cells**. **(a-h) **WMISH of *Smed-nb.21.11e *(a,c,e,g) and *Smed-agat-1 *(b,d,f,h) in *control(RNAi) *(a,b) and *Smed-H2B(RNAi) *animals 1 (c,d), 3 (e,f) and 5 (g,h) days after RNAi. Only one *control(RNAi) *time point (5 days) is shown since no differences were detected among them. Increased numbers of *Smed-nb.21.11e*- (c) and *Smed-agat-1*-positive cells (d) were detected 1 day after RNAi, with a subsequent decrease in numbers for both markers (e-h). **(i) **Quantification of the level of expression by qRT-PCR of *Smed-nb.21.11e *and *Smed-agat-1 *in *Smed-H2B(RNAi) *animals 1, 3 and 5 days after RNAi, normalized expression and relative to *control(RNAi) *samples. An upregulation of *Smed-nb.21.11e *and *Smed-agat-1 *transcript expression was detected 1 day after RNAi, with a subsequent decrease in levels for both transcripts. (a-h) Anterior is to the left. Scale bars: 500 μm.

We then wanted to analyze the early dynamics of progeny upon *Smed-H2B *RNAi by WMISH (Figure 5a-h). These analyses uncovered a peak in both early and late progeny categories by day 1 after RNAi. Numbers of both *Smed-nb.21.11e*- and *Smed-agat-1*-positive cells were increased by day 1 (Figure 4c,d versus Figure 4a,b), but started decreasing in numbers soon after. By day 3 (Figure 5e,f) and day 5 (Figure 5g,h) the numbers of *Smed-nb.21.11e*- and *Smed-agat-1*-positive cells were decreased below numbers found in *control(RNAi) *animals. In order to confirm this early peak in progeny cells, we performed qRT-PCR experiments (Figure 5i). These experiments confirmed that the amount of total transcripts from both *Smed-nb.21.11e *and *Smed-agat-1 *were increased by day 1, and decreased by day 3 and day 5, reaching about 20% for *Smed-nb.21.11e *and 50% for *Smed-agat-1*. Taken together, these experiments show that after *Smed-H2B *RNAi knockdown, the number of *Smed-nb.21.11e*- and *Smed-agat-1*-positive progeny cells first peak and later decrease in numbers. The differential behavior of NB and progeny cell markers upon *Smed-H2B *RNAi knockdown could be useful in differentiating between genes expressed in these different cellular compartments, both depleted by irradiation.

Taken together, these results show that *Smed-H2B *RNAi removes NBs, but at a slower pace than irradiation. Therefore, *Smed-H2B *RNAi can be used to characterize the expression profile of NBs, with potential advantages over irradiation. Firstly, *Smed-H2B *is expressed specifically in NBs, and therefore specifically affects these cells and their recent progeny and not differentiated cell types. Irradiation targets not only NBs but all other cell types, and its effects can be expected to induce organism-wide transcriptomic changes. Secondly, the dynamics of NB and progeny cells are different upon *Smed-H2B *RNAi and this may allow further resolution of the transcriptome profile of NB-specific and early progeny-specific genes, which are both rapidly downregulated upon irradiation.

### RNA-seq of irradiated and *Smed-H2B(RNAi) *samples

We decided to use the potent and specific *Smed-H2B *RNAi in order to clarify the transcriptional profiles of NBs. In order to leverage the expression profile dynamics described, we performed RNA-seq in RNA samples obtained from *Smed-H2B(RNAi) *worms 1, 2 and 5 days after RNAi, as well as *control(RNAi) *worms 5 days after injection. We also performed parallel RNA-seq experiments in irradiated samples 2, 4 and 7 days after irradiation, as well as non-irradiated controls (see Additional file 5 for summary). The reads from all samples were mapped to our reference transcriptome [9] and reads per kilobase mapped (RPKM) values for each transcript were calculated [45] and *control(RNAi) *and non-irradiated samples were subject to a low expression filter, leaving 17,262 transcripts with significant expression values across *control(RNAi) *and non-irradiated samples (Additional file 6).

**Figure 6:**
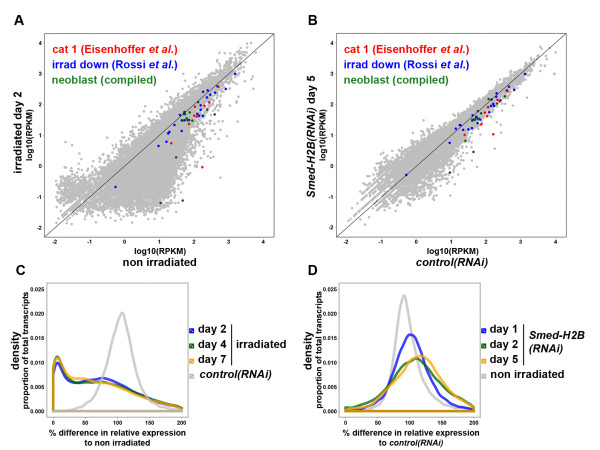
**RNA-seq of irradiated and *Smed-H2B(RNAi) *samples**. **(a,b) **Scatter plots of log10(RPKM) obtained for every transcript in irradiated day 2 versus non-irradiated samples (a) and *Smed-H2B(RNAi) *day 5 versus *control(RNAi) *samples (b). Category 1 (cat 1) genes described by Eisenhoffer and co-workers [24] are displayed in red, transcripts downregulated by irradiation described by Rossi and co-workers [25] in the planarian species *Dugesia japonica *are displayed in blue, and well known NB markers compiled from the literature are displayed in green. The scatter plot of irradiated versus non-irradiated samples is broader than that of *Smed-H2B(RNAi*) samples versus *control(RNAi)*, indicating more drastic differences in relative gene expression. However, most colored transcripts lay below the diagonal, indicating their downregulation in both NB ablation approaches. **(c) **Density plot of percentage of relative expression compared to non-irradiated samples of irradiated samples 2, 4 and 7 days after irradiation (colored lines), and *control(RNAi) *samples (grey line). The differences in relative gene expression between irradiated samples and non-irradiated are very broad, with many transcripts being downregulated to 0 to 10% of their relative expression in non-irradiated samples. **(d) **Density plot of percentage of relative expression compared to *control(RNAi) *samples of *Smed-H2B(RNAi) *samples 1, 2 and 5 days after RNAi (colored lines), and non-irradiated samples (grey line). The differences in relative gene expression between *Smed-H2B(RNAi) *samples and *control(RNAi) *samples are narrower, with the majority of transcripts still presenting around 100% of their relative expression in non-irradiated samples.

Comparison of irradiated and non-irradiated transcriptomes demonstrates that irradiation induces downregulation of a very large number of transcripts (Figure 6a) while *Smed-H2B(RNAi) *samples 5 days after RNAi versus *control(RNAi) *samples suggest an increased specificity of *Smed-H2B *RNAi, with most transcripts not changing in expression (Figure 6b). In order to see if the expression of genes expressed in NBs was downregulated in our samples, we compiled three different lists of genes. We mapped to our reference transcriptome the genes described as category 1 markers by Eisenhoffer and co-workers [24] (Additional file 7), genes downregulated by irradiation in the planarian species *Dugesia japonica *by Rossi and co-workers [25] (Additional file 8), and a list of known NB markers from the literature (Additional file 9). When we plotted the corresponding transcripts in our datasets most of them were found to be below the diagonal (Figure 6a,b), indicating that they were downregulated in both irradiated samples and *Smed-H2B(RNAi) *worms.

In order to further characterize differences between irradiation and *Smed-H2B *RNAi, we generated density plots of transcriptome-wide differential expression caused by either treatment. We plotted the proportion of transcripts against percentage change in expression compared to the respective control (Figure 6c,d, colored lines). We also plotted the difference in relative expression between the control samples of each parallel sequencing experiment (Figure 6c,d, grey lines). We found that the two control samples, non-irradiated worms and *control(RNAi) *worms, do not differ significantly, showing a normal distribution around no difference in expression. In contrast, the differences shown by irradiated samples when compared to non-irradiated worms were drastic, with a large proportion of transcripts in all three irradiated samples showing 0 to 10% of their normal expression in the control sample (Figure 6c, colored lines). When we generated a similar plot for *Smed-H2B(RNAi) *samples compared to *control(RNAi) *samples, we observed that the differences over the whole transcriptome were much smaller (Figure 6d, colored lines). Most transcripts still accumulate around 100% of the expression in *control(RNAi) *samples, indicating no change in gene expression for these transcripts, but a progressively flattened profile is observed as a significant number of gene expression level changes as NBs are depleted. The observed profiles reflect both loss of NBs but also relative proportional increases of genes expressed in the remaining differentiated cells. These data clearly support the notion of increased specificity of *Smed-H2B *RNAi as a method for genetic ablation of NBs.

### Dynamics of progeny transcripts in irradiated and *Smed-H2B *RNAi samples

**Figure 7:**
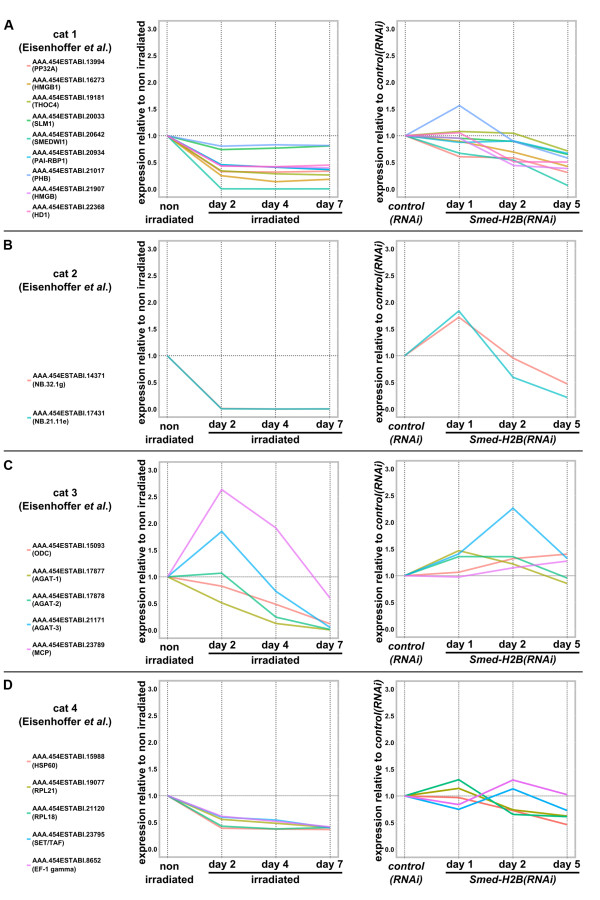
**Dynamics of category 1 to 4 markers in irradiated and *Smed-H2B(RNAi) *samples**. **(a-d) **Dynamics of category 1 (a), 2 (b), 3 (c) and 4 (d) transcript expression in irradiated samples (left) and *Smed-H2B(RNAi) *samples (right) relative to non-irradiated (left) and *control(RNAi) *(right) samples. All categories of transcripts downregulated by irradiation described by Eisenhoffer and co-workers [24] are consistently downregulated by day 7 after irradiation in our dataset (right). Only category 1 markers (a, right) are dowregulated progressively without peaking at any time point after *Smed-H2B *RNAi. Category 2 and 3 transcripts display a peak in *Smed-H2B(RNAi) *samples and category 4 transcripts are not downregulated as a whole.

In 2008, Eisenhoffer and co-workers [24] performed microarray analyses of gene expression after irradiation and categorized the genes found to be downregulated into four categories according to their expression patterns in irradiated and non-irradiated organisms. Category 1 transcripts were expressed in NBs, category 2 and 3 genes were found to be markers of the post-mitotic NB progeny, and category 4 genes were found to have a broad parenchymatic expression pattern. We mapped these markers to our transcriptome (Additional file 10) and investigated their dynamics after irradiation and *Smed-H2B *RNAi. Consistent with the study by Eisenhoffer and co-workers, all transcripts belonging to each of the four categories were downregulated by day 7 of irradiation (Figure 7a-d, left panels). Interestingly, though, the four different categories behaved differently in *Smed-H2B(RNAi) *samples. Category 1 genes, described to be genes expressed in NBs, were progressively downregulated in the different *Smed-H2B(RNAi) *samples (Figure 7a, right panel), consistent with our WMISH and qRT-PCR data for the transcripts described in Figure 4. Furthermore, none of these genes were upregulated at any of the time points of *Smed-H2B *RNAi, with the exception of the transcript AAA.454ESTABI.21017 (corresponding to the category 1 marker *prohibitin*; Additional file 11), which peaked at day 1 after *Smed-H2B *RNAi. When we analyzed the expression dynamics of category 2 and 3 markers we found that all of them were upregulated in at least one of the *Smed-H2B(RNAi) *samples, in agreement with our WMISH and qRT-PCR data for *Smed-nb.21.11e *and *Smed-agat1 *(Figure 5). Therefore, the peaking behavior of all progeny genes can be interpreted as a characteristic behavior of transcripts expressed in NB progeny upon NB ablation by *Smed-H2B *RNAi. Surprisingly, some category 3 transcripts also peaked on day 2 after irradiation (Figure 7c, left panel), suggesting that peaking behavior can apply to both irradiation and *Smed-H2B *RNAi. Interestingly, the transcript with a most prominent peak in day 2 after irradiation (corresponding to the progeny marker MCP; Additional file 11) is progressively upregulated in *Smed-H2B(RNAi) *samples, suggesting that its peak in *Smed-H2B *RNAi occurs later than 5 days after RNAi. Lastly, we compared the expression dynamics of category 4 genes in *Smed-H2B *RNAi. All of them were downregulated in all irradiated samples (Figure 7d, left panel), even though they were not found to have a NB-specific expression pattern by WMISH [24], and therefore can be considered as false positives of the irradiation approach. When we examined the expression dynamics of these transcripts in *Smed-H2B(RNAi) *samples we observed that they were not up- or downregulated as a whole (Figure 7d, right panel).

To investigate if the peak observed for progeny transcripts allowed us to predict new progeny markers, we performed WMISH in non-irradiated and irradiated animals for several transcripts that peaked in *Smed-H2B(RNAi) *samples 1 day after RNAi (Additional file 11). We found that two of them (AAA.454ESTABI.18948, encoding the novel *Smed-argininosuccinate-synthase*, and AAA.454ESTABI.18310, encoding the novel *Smed-soxF*) had expression profiles similar to progeny transcripts, and their expression did not disappear completely 48 hours after irradiation (Additional file 11). Another transcript (AAA.454ESTABI.16120, encoding the novel *Smed-histone-H1-gamma*) did have *in situ *expression pattern dynamics similar to NBs and disappeared completely after 48 hours. This latter transcript, with gene expression dynamics similar to the category 1 marker *prohibitin *(mentioned above), indicates that some genes with a NB expression pattern still peak after *Smed-H2B *RNAi. This probably reflects that both genes have an expression pattern that encompasses NBs and early postmitotic progeny.

Taken together, these results show that while irradiation induces downregulation of a large number of transcripts expressed in NBs and elsewhere, *Smed-H2B *RNAi induces a progressive and more specific downregulation of transcripts expressed in NBs, with genes expressed elsewhere behaving differently in the *Smed-H2B(RNAi) *dataset. Therefore, *Smed-H2B *RNAi can be used to describe the transcriptomic profile of NBs with increased specificity compared to irradiation.

### Extraction of transcripts confidently expressed in NBs by a combined irradiation and *Smed-H2B *RNAi approach

We then wanted to use our *Smed-H2B(RNAi) *dataset to compile a list of transcripts putatively expressed in NBs using overlap with our irradiation dataset to increase the reliability of our list. In order to do so, we first extracted lists of transcripts downregulated by both approaches separately. We selected transcripts that were downregulated in all three irradiated samples to at least 75% of their normal expression level in non-irradiated samples, and transcripts that were downregulated in *Smed-H2B(RNAi) *5 days after RNAi to at least 75% of their normal expression level in *control(RNAi) *samples. Furthermore, transcripts that peaked to more than 110% of their expression level in *control(RNAi) *samples were discarded in order to select against the inclusion of transcripts expressed in NB progeny.

**Figure 8:**
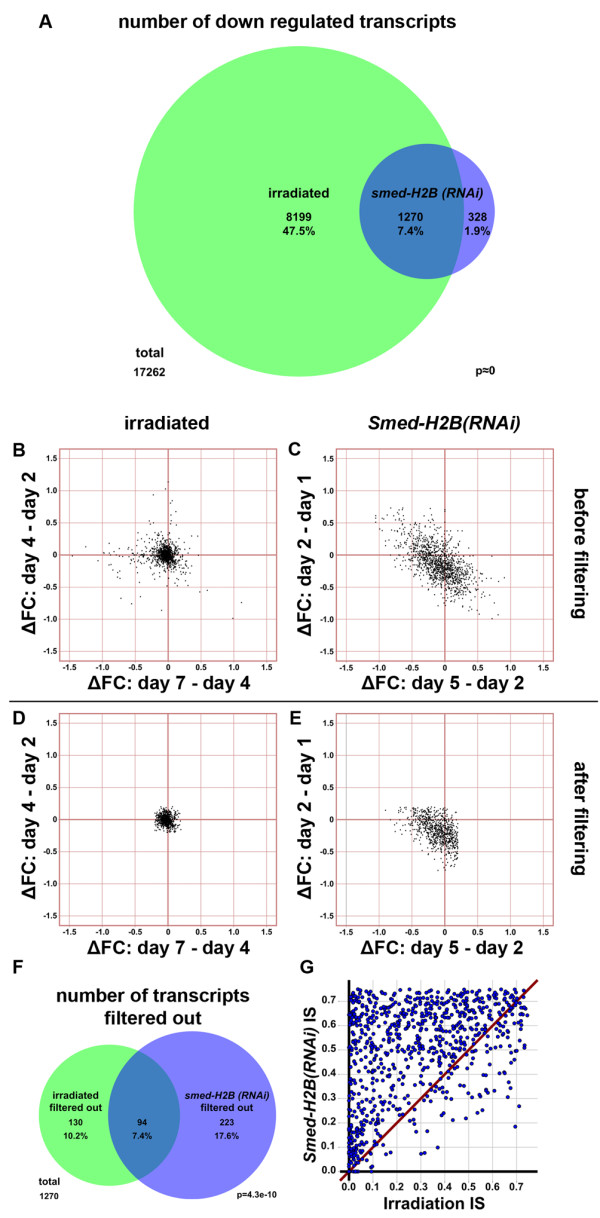
**Extraction of transcripts confidently expressed in NBs by a combined irradiation and *Smed-H2B *RNAi approach**. **(a) **Venn diagram of the number of transcripts downregulated in irradiated (green) and *Smed-H2B(RNAi) *(blue) samples. Irradiation downregulates more than half of our dataset of 17,262 transcripts. *Smed-H2B(RNAi) *downregulates less than 10% of the total number of transcripts. **{AU query: preceding sentence OK as edited?} YES **Most transcripts downregulated by *Smed-H2B *RNAi are also downregulated in irradiated samples (*P *≈ 0). **(b-e) **Δ Fold changes (ΔFC) between consecutive time points of irradiated (b,d) and *Smed-H2B(RNAi) *(c,e) samples. Most transcripts in irradiated samples tend to accumulate at the axis of the plot, indicating that their relative expression values in the three time points are similar (b). Most transcripts in *Smed-H2B(RNAi) *samples tend to accumulate in the bottom left quadrant, indicating that their relative expression values tend to be progressively lower (c). After filtering of transcripts that have fold changes of more than 0.2 or -0.2 in consecutive irradiation samples and of more than 0.2 in consecutive *Smed-H2B(RNAi) *samples (d,e), most transcripts adjust to the predicted behavior of NB-expressed transcripts. **(f) **Venn diagram of the number of transcripts filtered out by this filtering step in irradiated (green) and *Smed-H2B(RNAi)*. The relatively small *P*-value indicates that the overlap is significant. Transcripts filtered out by any of the filters were discarded in the other dataset as well. **(g) ***Smed-H2B(RNAi) *IS versus irradiation IS plot of the 823 transcripts not filtered out. Most of the transcripts are distributed above the diagonal, indicating that their *Smed-H2B(RNAi) *insensitivity (IS) is higher than their irradiation IS.

The results of this selection of transcripts are summarized in Figure 8a. A total of 9,469 transcripts were found downregulated by irradiation, but 8,169 of them were not downregulated by *Smed-H2B *RNAi. Conversely, 1,598 transcripts were found downregulated by *Smed-H2B *RNAi, with 1,270 of these transcripts (79.4%) also downregulated in the irradiation dataset. This high overlap (*P *≈ 0) indicates that the transcripts obtained by the two methods are highly correlated, with *Smed-H2B *RNAi yielding a more specific result when compared to irradiation. Irradiation downregulates more than half of the planarian transcriptome using simple criteria (54.9%). Of these, 8,199 transcripts (47.5%, constituting 86.6% of the transcripts downregulated after irradiation) are not downregulated after *Smed-H2B *RNAi, and are therefore unlikely to be expressed in NBs. Conversely, *Smed-H2B *RNAi downregulates a smaller number of genes, but most of them are found to be downregulated in irradiation. These results further prove the increased specificity of our *Smed-H2B *RNAi approach.

We generated a list with the 1,270 overlapping transcripts (Additional file 12), which amounts to 7.4% of our filtered transcriptome. We compared the behavior of these transcripts in more detail to the experimental genes examined (Figure 4) and the nine category 1 transcripts previously described (Figure 7a). We plotted the differential fold change between the different time points of irradiation or *Smed-H2B *RNAi (Figure 8b,c). As has been seen, the expression of category 1 transcripts tends to drop by day 2 of irradiation and then remain stable at the two later time points (Figure 7a). When we plotted the differences between irradiation time points, most transcripts fell close to the axis (Figure 8b), indicating that both the differences between day 4 and day 2, and between day 7 and day 4 of irradiation tend to be minimal, confirming that our 1,270 overlapping transcripts tend to behave like category 1 transcripts. Also, transcripts expressed in NBs tend to be progressively downregulated in *Smed-H2B(RNAi) *samples (Figures 4 and 7a). This behavior is also seen in our list of 1,270 transcripts (Figure 8c), with most transcripts falling in the bottom left quadrant, indicating that their relative expression decreases both from day 1 to day 2 and from day 2 to day 5 of *Smed-H2B *RNAi (Figure 8c). However, a considerable number of transcripts still fluctuated away from these predicted behaviors. In order to more accurately filter our dataset, we employed an extra, more stringent filtering step, which eliminated all transcripts with differences higher than 0.2 (20% of the expression in non-irradiated samples) in the irradiation dataset and with increases in expression higher than 0.2 (20% of the expression in *control(RNAi) *samples) between any of the *Smed-H2B *RNAi successive time points. The result of this filtering step can be seen in Figure 8d,e. After this strict filtering step, 823 transcripts remained (Additional file 13) that show behavior similar to transcripts expressed in NBs, as seen in Figures 4 and 7a. The transcripts filtered out by the described filter also overlapped significantly (*P*-value = 2.70e-10), with 94 transcripts (of a total of 447) filtered out by both methods (Figure 8f). These filtered transcripts may represent transcripts that are expressed in NBs (as expression is reduced by ablation) but also other cells where expression responds dynamically to NB ablation.

### Approximation of the level of NB ablation insensitivity for each transcript

We then wanted to compare the amount of expression left after NB ablation by both methods for every transcript. As shown in Figure 4, after NB ablation, transcripts that are expressed only in NBs have low relative expression levels, while the expression of transcripts that are also detected in the CNS is still present at levels 40 to 70% of those in control samples, reflecting the irradiation- or *Smed-H2B *RNAi*-*insensitive proportion of expression. This insensitive proportion is therefore indicative of the amount of transcripts that do not localize to NBs, mostly detected in the CNS (Figure 4). To perform this comparison on our list of transcripts, we calculated the expression left after irradiation by averaging the relative expression values of all three irradiated samples, since this amount tends to be stable at all three time points. We called this parameter 'irradiation insensitivity' (irradiation IS). We also used the expression values 5 days after *Smed-H2B *RNAi as indicative of the proportion of expression insensitive to *Smed-H2B *RNAi, and called this value '*Smed-H2B(RNAi) *insensitivity' (*Smed-H2B(RNAi) *IS). These values are given for each transcript in Additional file 13. After a first inspection of these values we found that the transcripts previously described to be expressed only in NBs had consistently low values for both of these parameters (0.00 and 0.07, respectively, for AAA.454ESTABI.22122, encoding *Smed-pcna*, and 0.00 and 0.07, respectively, for AAA.454ESTABI.20642, encoding *Smedwi-1*), indicating that most of their expression was eliminated by both irradiation and *Smed-H2B *RNAi, in agreement with our other experimental data. Consistently, the transcripts with prominent expression in the CNS had higher values for both parameters (0.31 and 0.71, respectively, for AAA.454ESTABI.16133, encoding *Smedtud-1*, and 0.10 and 0.53, respectively, for AAA.454ESTABI.9082, encoding *Smedwi-2*). In order to confirm the agreement between these two methods, we plotted the values obtained by both methods for each transcript (Figure 8g). A high correlation was expected, with most dots falling near the diagonal. However, the actual distribution differed dramatically from this expectation. Most dots are distributed above the diagonal towards the top left of the graph, with only a very few transcripts below the diagonal. This shows that insensitivity to irradiation and *Smed-H2B *RNAi are not equivalent but nonetheless still display a relationship. The observed distribution shows that irradiation IS tends to be smaller than *Smed-H2B(RNAi) *IS. This distribution suggests that the proportion of expression left after irradiation (and reflected by our parameter 'irradiation IS') is greatly affected by the irradiation across the transcriptome as a whole, and that irradiation downregulates the expression of NB-expressed transcripts in the other cell populations in which they are also expressed. Therefore, we consider that *Smed-H2B(RNAi) *IS constitutes a better approximation of the relative expression that is insensitive to NB ablation and remains in other cell types, and that the distribution of the two IS parameters results from another general artifact introduced by irradiation treatment.

### Validation of NB-expressed transcripts

**Figure 9:**
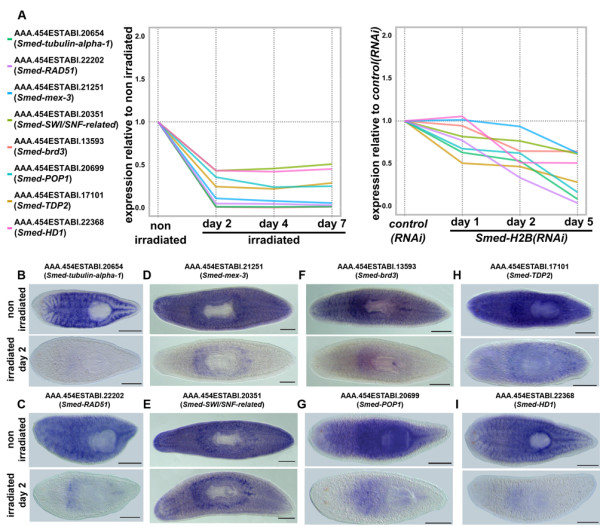
**Validation of NB-expressed transcripts**. **(a) **Dynamics of validated transcripts expression in irradiated samples (left) and *Smed-H2B(RNAi) *samples (right) relative to non-irradiated (left) and *control(RNAi) *(right) samples. **(b-i) **WMISH of the transcripts AAA.454ESTABI.20654 (*Smed-tubulin-alpha-1*) (b), AAA.454ESTABI.22202 (*Smed-RAD51*) (c), AAA.454ESTABI.21251 (*Smed-mex-3*) (d), AAA.454ESTABI.20351 (*Smed-SWI/SNF-related*) (e), AAA.454ESTABI.13593 (*Smed-brd3*) (f), AAA.454ESTABI.20699 (*Smed-POP1*) (g), AAA.454ESTABI.17101 (*Smed-TDP2*) (h) and AAA.454ESTABI.22368 (*Smed-HD1*) (i) in non-irradiated animals (top panels) and animals 2 days after irradiation (bottom panels). All validated transcripts, including the category 1 marker *Smed-HD1*, show a NB expression pattern that disappears in irradiated animals.

We then wanted to experimentally validate our list of 823 transcripts putatively expressed in NBs. We picked previously undescribed transcripts from our list together with another control category 1 gene (Figure 9a) and performed WMISH in non-irradiated and irradiated samples 2 days post-irradiation (Figure 9b-i). All genes showed a NB-specific staining pattern, with some of them displaying detectable expression in the CNS. Interestingly, no expression in the CNS was observed for AAA.454ESTABI.20654, encoding the NB-specific *Smed-tubulin-alpha-1 *(Figure 9b). The values of the irradiation IS and *Smed-H2B(RNAi) *IS parameters were low for this transcript, as would be expected of a transcript expressed only in NBs and not elsewhere (0.01 and 0.08, respectively; Additional file 13).

### Annotation of our list of NB-expressed transcripts

We then wanted to investigate the nature of the transcripts obtained by our approach. In order to do that, we obtained BLAST hits [46] and GO terms [47] for each of the transcripts, looked for conserved protein domains encoded by them and established KEGG annotations for them using the KAAS application [48,49]. These analyses were performed in parallel with our initial filtered transcriptome list of 17,262 in order to provide comparisons and enrichment analyses. The results of this annotation are summarized in Additional files 14 to 16.

First, we wanted to see if differentiated cell markers were present in our list. We looked at our KEGG annotations (Additional file 16) and saw that pathways present *a priori *in differentiated cells were not enriched in our dataset. Furthermore, even though our list contains transcripts expressed in the CNS and NBs, CNS-specific transcripts were not present in our list either. For instance, no signs of enrichment were found for the KEGG pathways 'Axon guidance' (*P *= 1), 'Neurotrophin signaling pathway' (*P *= 1), or 'Long term potentiation' (*P *= 1).

### NB-expressed transcripts reveal the molecular detail of morphological observations from NBs

**Figure 10:**
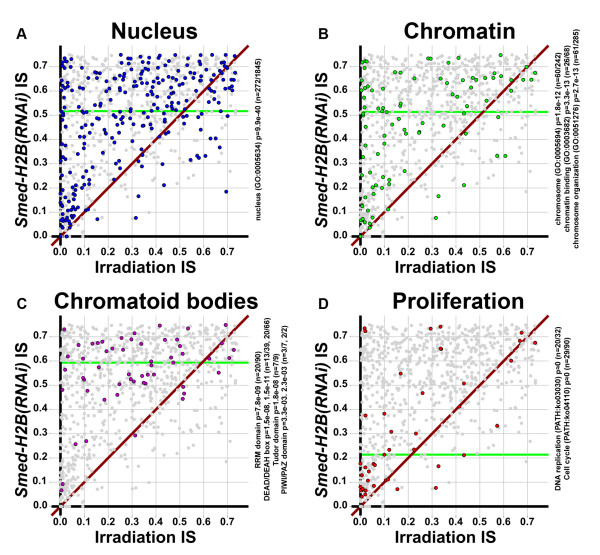
**NB-expressed transcripts reveal the molecular detail of morphological observations from NBs**. **(a-d) ***Smed-H2B(RNAi) *IS versus irradiation IS plot of the transcripts compiled with the GO term 'nucleus' (a, blue), the GO terms 'chromosome', 'chromatin binding' and 'chromosome organization' (b, green), the presence of RNA recognition motif, DEAD/DEAH helicase, Tudor and PIWI/PAZ domains (c, yellow), and the KEGG pathways 'DNA replication' and 'Cell cycle' (d, red). Green horizontal lines represent the median *Smed-H2B(RNAi) *IS of each compilation. Our list of NB-expressed transcripts is enriched in nuclear components (a), chromatin associated components (b), RNA binding and putative RNA granule components (c) and cell division machinery (d). Nuclear components (a) and chromatin-associated components (b) tend to be distributed all over the plot. However, putative CB components (c) tend to be distributed in the upper part of the plot, suggesting expression outside of the NB compartment, while cell division machinery components (d) tend to be distributed close to the axis, indicating their expression in NBs exclusively.

We found that our list of NB cell transcripts was consistent with the classical morphological observations of NBs. First, we saw that the GO term 'nucleus' was highly enriched in our list (Figure 10a; Additional file 14), and consistent with the high nucleus versus cytoplasm ratio of NBs. The transcripts annotated with the GO term 'nucleus' distributed all along the *Smed-H2B(RNAi) *IS versus irradiation IS plot, indicating that the GO term 'nucleus' is enriched in both transcripts expressed in NBs and transcripts that are also expressed elsewhere, probably in the CNS.

Next, we generated a set of genes by combining the transcripts annotated with the GO terms 'chromosome', 'chromatin binding' and 'chromosome organization' (Figure 10b; Additional file 17), which were also enriched in our list. This enrichment indicates that the classical morphological observation of open chromatin translates at the molecular level into a high occurrence of transcripts that drive chromatin-associated processes. This list contains transcripts encoding elements of the DNA replication machinery (Additional file 17), but also histone-modifying factors such as a histone acetyl transferase (AAA.454ESTABI.22989), a histone deacetylase (AAA.454ESTABI.22368; see also Figure 9), a mll5 protein (AAA.454ESTABI.12992), several chromodomain-containing proteins, including the previously described *Smed-CHD4 *(AAA.454ESTABI.22442) [21], and other chromatin remodeling enzymes such as a Suppressor of Zeste homolog (AAA.454ESTABI.20203).

We then wanted to check the prevalence of putative CB components in our list. In order to do so we searched for domains (Additional file 15) known to be involved in RNA granules and germ granules [50]. We compiled a list of transcripts that encode RNA recognition motif (RRM) domains, DEAD/DEAH-box domains, Tudor domains and PIWI/PAZ domains (Figure 10c; Additional file 18). These domains were found to be highly enriched in our list (see Additional file 15 and Figure 10c for *P*-values). RRM domains are RNA interacting domains that are known to be present in proteins such as Bruno proteins [51,52]. The previously described Bruno homolog of *S. mediterranea *[17] was identified in this list (AAA.454ESTABI.20564). Also, we looked for transcripts encoding either DEAD/DEAH box helicase domains or the helicase carboxy-terminal domain, which are both present in DEAD/DEAH box RNA helicases [53]. We found 24 transcripts encoding either one of these domains, with nine of them encoding both. Among these proteins we found a ddx4/Vasa homolog (AAA.454ESTABI.22361), a classical component of germ granules [54], and homologs for several other DEAD box RNA helicases (see Additional file 18 for a detailed list). Significantly, when we looked for transcripts encoding Tudor domain-containing proteins [55,56], we found seven transcripts out of a total of nine found in our background transcriptome, again constituting a significant enrichment (*P *= 1.8e-08). This list contains *Smedtud-1 *(AAA.454ESTABI.16133), which was described to be a CB component in *S. polychroa *[19], as well as six other transcripts encoding Tudor domain-containing proteins. Finally, we looked for transcripts encoding either PIWI or PAZ domains, since they have been functionally linked with RNA granules in several organisms [57]. With this approach we recovered four transcripts, encoding *Smedwi-1 *(AAA.454ESTABI.20642), *Smedwi-2 *(AAA.454ESTABI.9082 and AAA.454ESTABI.14275) and *Smedwi-3 *(AAA.454ESTABI.17140).

Interestingly, most of the transcripts compiled in this list distributed to the upper part of the *Smed-H2B(RNAi) *IS versus irradiation IS plot (Figure 10c), with the notable exceptions of AAA.454ESTABI.20642 (encoding *Smedwi-1*) and AAA.454ESTABI.19819 (annotated as the RNA-helicase ercc-6, involved in DNA excision repair). This distribution likely reflects the fact that most RNA-binding proteins and proteins expressed in CBs are also present in the planarian CNS, as has been seen for *Smedtud-1 *(Figure 4o). This notion implies that the distribution relies on the *Smed-H2B(RNAi) *IS value, and not irradiation IS, for which all these transcripts have random values. The fact that neurons also display RNA granules [27], which is where the *S. polychroa *homolog of *Smedtud-1 *is located [19], supports this hypothesis and suggests molecular and functional connections between NBs and neuronal cells.

We then wanted to check if the machinery of cell division was enriched in our list of NB-expressed transcripts. NBs are the only proliferative cell type of asexual planarians [23,58], and it is expected, therefore, that a significant part of their transcriptome will encode for the cellular machinery driving proliferation. In order to do so, we compiled a list with our KEGG orthology map, corresponding to KEGG pathways 'DNA replication' and 'Cell cycle' (Additional file 19), and plotted these transcripts' *Smed-H2B(RNAi) *IS versus irradiation IS values (Figure 10d). Most of these transcripts distribute near the axis of the plot, reflecting that they have low values for both plotted parameters. This indicates that most of these transcripts are not expressed in the CNS or elsewhere, but confined to NBs. This is consistent with the fact that NBs are the only proliferative cell type of asexual planarians and validates our approach for discriminating transcripts expressed in NBs from transcripts that are shared by NBs, neurons and possibly other cell types. This list includes transcripts encoding genes such as *Smed-pcna *(AAA.454ESTABI.22122) [39] and *Smed-mcm2 *(AAA.454ESTABI.20533) [40], and several other transcripts encoding cellular division machinery proteins, with most of them having low *Smed-H2B(RNAi) *IS and irradiation IS (Additional file 19).

Taken together, these observations validate our approach by showing that most of the morphological features of NBs are translated to the molecular level in our datasets. A strong enrichment of nuclear components, chromatin remodeling factors, RNA binding proteins and other RNA granule-related components and cell division machinery is found in our list of NB-expressed transcripts.

### Regulation of NBs heavily relies on posttranscriptional gene regulation mechanisms

**Figure 11:**
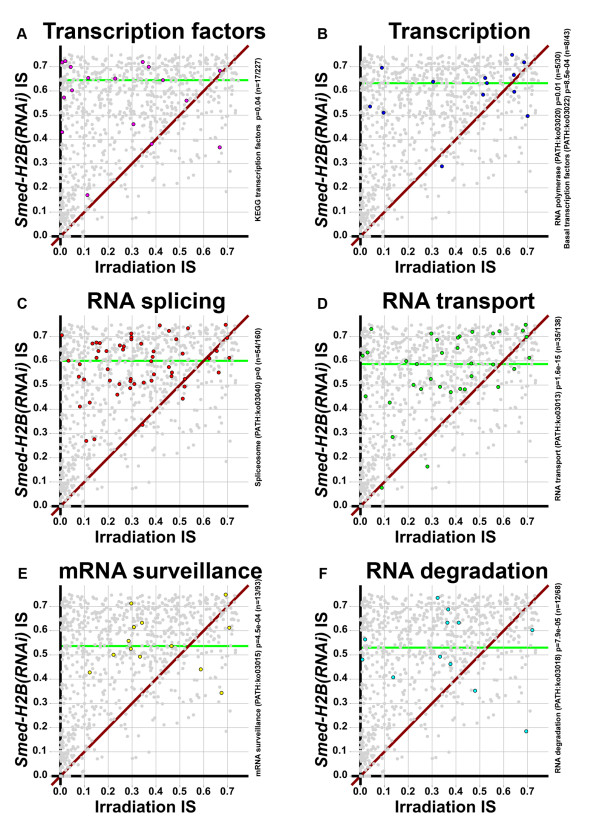
**Regulation of NBs relies heavily on PTGR mechanisms**. **(a-f) ***Smed-H2B(RNAi) *IS versus irradiation IS plot of the transcripts compiled with KEGG (BRITE hierarchies) transcription factors (a, magenta), KEGG pathways 'RNA polymerase' and 'Basal transcription factors' (b, blue), KEGG pathway 'Spliceosome' (c, red), KEGG pathway 'RNA transport' (d, green), KEGG pathway 'mRNA surveillance' (e, yellow) and KEGG pathway 'RNA degradation' (f, cyan). Green horizontal lines represent the median *Smed-H2B(RNAi) *IS of each compilation. Several transcription factors are found in our list of NB-expressed transcripts, but they are not significantly enriched (a), and similarly, transcription machinery is not significantly enriched either (b). However, a strong enrichment is found for spliceosome components (c). Other RNA-associated processes, such as RNA transport (d), mRNA surveillance (e) and RNA degradation (f), are enriched as well with various significances.

KEGG annotations of our list of NB-expressed transcripts showed a strong enrichment of the KEGG pathways 'RNA splicing' and 'RNA transport' (Additional file 16), indicating that PTGR [59] plays a fundamental role in NB biology. In order to further look at this question, we compiled lists of transcripts using our KEGG annotations encoding transcription factors (Figure 11a; Additional file 20), transcription machinery (Figure 11b; Additional file 21), RNA splicing (Figure 11c; Additional file 21), RNA transport (Figure 11d; Additional file 21), mRNA surveillance (Figure 11e; Additional file 21) and RNA degradation (Figure 11f; Additional file 21). We did not find a significant enrichment of transcription factors in our NB-expressed transcripts - only 17 of the 227 transcription factors annotated by KEGG. However, the rest of the lists compiled showed a significant enrichment, with our list of transcription machinery transcripts being the least significantly enriched (KEGG pathway ko03022, *P *= 0.01, KEGG pathway ko03022, *P *= 8.5e-04), and our list of RNA splicing transcripts (KEGG pathway ko03040, *P *≈ 0) being the most significantly enriched.

Among the transcription factors found (Figure 11a; Additional file 20), we found several general chromatin remodeling factors, like the above mentioned mll5 homolog (AAA.454ESTABI.12992), but also a Sox transcription factor (AAA.454ESTABI.19118) and a Y-box transcription factor (AAA.454ESTABI.17447). Most of these factors seem to be expressed in both NBs and other cells given the proportion of their expression not affected by NB ablation. The genes compiled in the transcription list (Figure 11B, also see Additional file 21) mostly include components of the transcription machinery, such as several components of the RNA polymerases I, II and III.

A surprising and previously undescribed enrichment of splicing machinery was found in our dataset, comprising several small nuclear ribonucleoproteins, RNA helicases and splicing factors (Additional file 21). These findings uncover that the regulation of splicing, which is a key stage of PTGR [59], is probably fundamental for NB biology, and highlight the importance of PTGR in NBs. Furthermore, a strong enrichment was also found for RNA transport (Figure 11d). The genes contained in this list include several factors involved in the export of mRNA from the nucleus to the cytoplasm, such as an exportin-1 homolog (AAA.454ESTABI.20917), and several transcripts encoding components of the nuclear pore, such as the nuclear pore complex protein nup160 (AAA.454ESTABI.24286). Interestingly, CBs have been traditionally associated with nuclear pores [13,60], and, overall, these observations link the enrichment of splicing machinery, RNA transport machinery and CBs and suggest a framework where CBs play a central role in orchestrating the regulation of gene expression in NBs, with a strong role for PTGR mechanisms.

We also found a significant enrichment of the KEGG pathways 'mRNA surveillance' (Figure 11e; Additional file 21) and 'RNA degradation' (Figure 11f; Additional file 21), which are intimately close to the previous ones [59], and which include components of the nonsense-mediated decay and other RNA quality surveillance mechanisms, such as an upf2 regulator of nonsense transcripts (AAA.454ESTABI.21804) and the exosome component 8 (AAA.454ESTABI.23262). These findings contribute to the integrative view of RNA based-PTGR control as fundamental for planarian stem cell biology.

## Discussion

In this study we introduce the use of *Smed-H2B *RNAi as a means for the genetic ablation of NBs. We demonstrate that it eliminates the expression of NB-expressed genes in a short period of time and use this in order to elucidate the transcriptional profiles of NBs. *Smed-H2B *RNAi induces a quick NB loss that happens in a period of 5 days, faster than most RNAi knockdowns of genes involved in NB biology. For instance, during *Smedwi-2 *RNAi, NBs and their mitotic activity are detected for more than 10 to 15 days [16], and similar periods were described for the gene knockdowns of *Smed-bruno-like *[17], *Smed-SmB *[61] and *Smed-CHD4 *[21]. The speed of the *Smed-H2B *RNAi phenotype makes this knockdown an excellent tool for the genetic ablation of NBs.

*Smed-H2B *is a NB-specific histone isoform that is likely needed for the progression of NBs through the S-phase of the cell cycle. Its RNAi-driven knockdown probably causes a cycling failure. It is still unknown if NBs die at this point or simply differentiate. The consistent peak in NB progeny markers [24] suggests that at least part of the NB population commits to differentiation, and this is what creates the peaking dynamics of category 2 and 3 markers. It is likely that this also happens after irradiation, since some progeny markers also display a prominent peak after irradiation. Some transcripts with a typical NB expression pattern were found to peak during *Smed-H2B *RNAi as well. This suggests that some of the expression of these transcripts is also localized to early NB progeny, and they are only downregulated later in the differentiation process, although further work is still needed in order to elucidate this question.

Our *Smed-H2B *RNAi approach for the discovery of transcripts expressed in NBs is very specific in comparison to irradiation. Irradiation is known to affect not only the proliferative cells, but to introduce DNA damage in all cells and therefore induce a very potent stress response. As a consequence of this, nearly half of our transcriptomic dataset is consistently downregulated upon irradiation, with a very significant portion of these transcripts not being NB specific. This fact was already observed by Eisenhoffer and co-workers [24], and solved by carefully checking the WMISH patterns of expression of the transcripts downregulated by irradiation. Our approach solves this question in a very specific way, since all categories described by Eisenhoffer and co-workers behave differently after *Smed-H2B *RNAi. Therefore, *Smed-H2B *RNAi is informative because of its specificity and discriminative power compared to irradiation.

Our RNA-seq quantification of transcripts expressed in NBs by irradiation and *Smed-H2B *RNAi reveals that a significant amount of the downregulated transcripts do not disappear after NB ablation, and therefore are expressed at significant levels outside of the NB cellular compartment. However, we show that the quantification of the amount of transcript insensitive to NB ablation differs significantly by both methods. Our analyses of this question indicates that these differences are likely an artifact of irradiation, since the values for irradiation tend to be lower but random when compared to the values determined with *Smed-H2B(RNAi)*. More precisely, for several of the functional gene sets that we describe through annotation of our data, the expression left after NB ablation is consistent for *Smed-H2B *RNAi but random for irradiation. This fact highly supports that our *Smed-H2B *RNAi approach is more successful in estimating the amount of transcript left after NB ablation and therefore the amount of expression outside of NB stem cells. Therefore, irradiation not only downregulates nearly half of the planarian transcriptome, but also downregulates the portion of expression left after NB ablation for a very large number of transcripts. The extent of this artifact can also vary according to the detection method used (qRT-PCR, WMISH or RNA-seq), therefore introducing confusion when elucidating the expression patterns of a very significant number of genes. Our *Smed-H2B *RNAi approach solves this problem and will therefore be a valuable tool for the functional studies of these transcripts and for NB ablation in the future.

The transcripts described as expressed in NBs by our combined approach recapitulate the known morphological features of NBs. A strong enrichment of nuclear components is found, reflecting the high nucleus versus cytoplasm ratio of planarian stem cells. A strong enrichment for chromatin remodeling factors and putative CB components is also found, paralleling the morphological observations of NB chromatin and CBs. Furthermore, a very prominent enrichment of the cell proliferation machinery is also found in our NB-expressed transcripts, agreeing with the fact that NBs are the only proliferative cell type of asexual planarians.

For most of the genes studied here and in the literature, transcripts that do not localize only in NBs are also localized in the planarian CNS [15,17,19-22,61]. Interestingly, neurons in other organisms also contain RNA granules, often called neuronal granules [62,63], which are similar at the morphologic and biochemical levels to other kinds of RNA granules [50,64,65], and are believed to have similar RNA processing roles. Neuronal RNA granules function in the translational repression and transport of nuclear mRNAs to their different destinations in order to be translated locally. Neuronal granules are found in a variety of organisms, and have been described as well in freshwater planarians [27]. Interestingly, the *S. polychroa *homolog of *Smedtud-1 *and the *D. japonica *protein *DjCBC-1 *have both been localized to perinuclear granules in NBs and neurons, further evidence of the amount of overlap that exists between the molecular machinery of CBs and neuronal granules in planarians. Our data reveal that this overlap is very substantial. Most of the NB-expressed transcripts detected by our approach have a significant portion of their expression that is insensitive to NB ablation. This fact suggests that they are likely present in the planarian CNS, and is consistent with the presence of neuronal granules in the planarian brain as well.

The most enriched process found in our dataset is RNA splicing, revealing that splicing must be of fundamental importance for the regulation of planarian stem cells. Significantly, the regulation of alternative splicing has recently been linked to stem cell biology and embryonic stem cells [66-72]. Our data uncover that splicing must also be of fundamental importance for the regulation of planarian stem cells and therefore suggest that it is a key conserved process in the regulation of stem cells.

Our data offer an integrated view, in which the PTGR control is fundamental for the regulation of both NBs and neurons, and in concert with chromatin remodeling and maintenance of the undifferentiated state [73]. RNA granules and the CBs of NBs are probably a fundamental hub for this control, integrating the processes of splicing and RNA transport, and other RNA related cell functions. CBs have been classically associated with nuclear pores from the morphological point of view. Our data are consistent with this observation, identifying several nuclear pore components as expressed in NBs, suggesting that CBs are the repository of nuclear transcribed mRNAs, where PTGR occurs. This regulation is probably affected by, and ultimately affects, the processes of chromatin remodeling [73]. Splicing and mRNA quality processes are also probably integrated in CBs. Overall, our NB ablation transcriptome approach reveals that regulation of planarian stem cells relies heavily on PTGR mechanisms.

## Conclusions

Here, we introduce a novel genetic knockdown method using *Smed-H2B(RNAi)*, a histone variant specifically expressed in NBs, and use this very specific method for investigating the transcriptome of planarian stem cells. Our experiments show that *Smed-H2B *RNAi knockdown induces a powerful and specific phenotype and that the use of this phenotype in order to perform RNA-seq studies to investigate NB expression profiles is more specific than the classical method of irradiation. We uncover a list of 823 transcripts confidently expressed in NBs and validate their expression profiles. Our data highlight the importance of posttranscriptional mechanisms in stem cell regulation, with an unexpectedly high enrichment of genes involved in RNA splicing and other posttranscriptional RNA-based processes, and no significant enrichment of transcriptional mechanisms. This knowledge is important for both planarian research and stem cell biology since many of the mechanisms that regulate stem cells are likely to be conserved.

## Materials and methods

### Organisms

Planarians of the asexual strain of *S. mediterranea *were kept and used as previously described [74].

### RNAi

RNAi experiments were carried out as previously described [74]. *Control(RNAi) *worms were injected with dsRNA encoding green fluorescent protein, a gene not present in the *S. mediterranea *genome. Essentially, animals were injected for three consecutive days. Day 1 after RNAi is considered to be the day after the third dsRNA injection. dsRNAs for *Smed-H2B *RNAi were *in vitro *transcribed from a PCR amplicon using the following *Smed-H2B *primers: 5'- TCTGTTAAGAAGATTTCAAAGG-3' and 5'- TCCTGTGTATTTTGTAACAGC-3'.

### Irradiation

Irradiation experiments were carried out as previously described [75]. Essentially, animals were administered at a dose of 100 Gy of gamma radiation using a sealed ^137^Cs source and fixed at different days after irradiation.

### *In situ *hybridization, immunohistochemistry and imaging

WMISH, FISH on histological sections and WMIHC were performed and imaged as previously described [19,74,76]. Anti-phospho-histone-3 (Millipore, Billerica, MA, USA) was used at a 1:500 dilution. Phospho-histone-3-positive cells were counted using ImageJ on confocal Z-stacks of whole mount immunostained animals.

### qRT-PCR

qRT-PCR experiments were performed as previously described [19], with modifications. Five animals were used per time point, replicate and treatment. Experiments were performed on three biological replicates per time point and treatment. Each biological replicate was technically replicated three times in each reaction, and each reaction was repeated three times. Results were normalized by the expression of the control housekeeping gene *Smed-ef2*, averaged and expression relative to *control(RNAi) *or non-irradiated samples presented.

### RNA-seq and analysis

Duplicate libraries for RNA-seq analysis were prepared from total RNA at the appropriate experimental time point of irradiation (2, 4 and 7 days) and for mock irradiated whole worms and sequenced with a SOLiD 4 sequencer (Lifetech, Paisley, United Kingdom), exactly as previously described [9]. Data were mapped and analyzed as previously described to produce RPKM values [9]. This procedure was repeated with batches of ten worms for *Smed-H2B *RNAi at 1, 2 and 5 days after the final RNAi injection and for *control(RNAi) *worms 5 days after the final injection. These time points were selected in light of our earlier *Smed-H2B *RNAi-mediated NB ablation. The results of these RNA-seq experiments have been deposited in the European Bioinformatics Institute under the accession ERP001079. RPKM values were filtered for transcripts that were expressed only at low levels (<10 mapped reads) and compared using simple PYTHON scripts available on request. KEGG annotations were assigned with KAAS [48,49]. GO and PFAM annotations were previously assigned [28] and analyzed for enrichment using the Ontologizer software package [77]. Significance values for domains and KEGG terms were calculated using the hypergeometric function in the R statistical package.

## Abbreviations

CB: chromatoid body; CNS: central nervous system; dsRNA: double-stranded RNA; FISH: fluorescent *in situ *hybridization; GO: Gene Ontology; IS: insensitivity; KEGG: Kyoto Encyclopedia of Genes and Genomes; NB: neoblast; PTGR: posttranscriptional gene regulation; qRT-PCR: quantitative RT-PCR; RNAi: RNA interference; RPKM: reads per kilobase mapped; RRM: RNA recognition motifs; Smed-H2B: Smed-histone-2B; WMIHC: whole mount immunohistochemistry; WMISH: whole mount *in situ *hybridization.

## Competing interests

The authors declare that they have no competing interests.

## Authors' contributions

JS conceived and designed the study, performed RNAi experiments and *in situ *hybridization/immunohistochemistry analyses, analyzed the data and wrote the manuscript. DK analyzed the data. YM and FJ-H performed validation *in situ *hybridizations. SM and RW prepared libraries for RNA-seq and performed RNA-seq experiments. AAA conceived and designed the study, analyzed the data and wrote the manuscript. All authors approved the final manuscript.

## Supplementary Material

Additional file 1 **Dynamics of *Smed-nb.21.11e*-positive cells after irradiation**. **(a-d) **WMISH of *Smed-nb.21.11e *in non-irradiated (a) and irradiated animals 1 (b), 3 (c) and 6 (d) days after irradiation. *Smed-nb.21.11e*-positive cells are still detected 1 day after irradiation, but strongly decline in numbers after 3 days (b) and are not detectable after 6 days (d). Anterior is to the left. Scale bars: 500 μm. Click here for file

Additional file 2 **Dynamics of *Smed-agat-1*-positive cells after irradiation**. **(a-d) **WMISH of *Smed-agat-1 *in non-irradiated (a) and irradiated animals 3 (b), 5 (c) and 10 (d) days after irradiation. *Smed-agat-1*-positive cells are depleted from the anterior region of the worm 3 days after irradiation, strongly decline in numbers after 5 days (b) and are not detectable after 10 days (d). Anterior is to the left. Scale bars: 500 μm. Click here for file

Additional file 3 **Dynamics of expression of *Smed-mcm2 *and *Smedwi-3 *in *Smed-H2B(RNAi) *animals**. **(a-h) **WMISH of *Smed-mcm2 *(a,c,e,g) and *Smedwi-3 *(b,d,f,h) in *control(RNAi) *(a,b) and *Smed-H2B(RNAi) *animals 1 (c,d), 3 (e,f) and 5 (g,h) days after RNAi. Most signals located in NBs disappear progressively for both markers (c-h). Almost no signals are detected 5 days after RNAi for the NB-specific marker *Smed-mcm2 *(g). The expression in the CNS of *Smedwi-3 *(h) is not eliminated by *Smed-H2B *RNAi and becomes more apparent after 5 days of RNAi (h). Some expression is detected in two rows of dorsal cells (g,h). Anterior is to the left. Scale bars: 500 μm. Click here for file

Additional file 4 **Dynamics of *Smed-nanos*-positive cells after *Smed-H2B *RNAi**. **(a-d) **WMISH of *Smed-nanos *in *control(RNAi) *(a) and *Smed-H2B(RNAi) *animals 1 (b), 3 (c) and 5 (d) days after RNAi. *Smed-nanos*-positive cells are distributed as two rows of NB-like dorsal cells, and are still detected, although severely reduced, 5 days after *Smed-H2B *RNAi (d). Anterior is to the left. Scale bars: 500 μm. Click here for file

Additional file 5 **Summary of mapped reads**. Click here for file

Additional file 6 **Transcriptomic data after low expression filter**. Click here for file

Additional file 7 **Mapping of category 1 transcripts described by Eisenhoffer and co-workers **[24]. Click here for file

Additional file 8 **Mapping of transcripts downregulated by irradiation by Rossi and co-workers **[25]. Click here for file

Additional file 9 **Mapping of known neoblast markers compiled from the literature**. Click here for file

Additional file 10 **Category 1, 2, 3 and 4 transcripts described by Eisenhoffer and co-workers **[24]. Click here for file

Additional file 11 **Validation of peaking transcripts**. **(a) **Dynamics of validated peaking transcript expression in irradiated samples (left) and *Smed-H2B(RNAi) *samples (right) relative to non-irradiated (left) and *control(RNAi) *(right) samples. **(b-d) **WMISH of the transcripts AAA.454ESTABI.18948 (*Smed-argininosuccinate-synthase*) (b), AAA.454ESTABI.18310 (Smed*-soxF*) (c) and AAA.454ESTABI.16120 (*Smed-histone-H1-gamma*) (c) in non-irradiated and animals 2, 4 and 7 days after irradiation. *Smed-argininosuccinate-synthase *(b) and *Smed-soxF *(c) signals are lost progressively after irradiation, in a pattern similar to progeny markers. *Smed-histone-H1- gamma *signals are distributed in a pattern reminiscent of NBs and the majority of signals are lost after 2 days of irradiation. Click here for file

Additional file 12 **Combined list of 1,270 transcripts downregulated in both irradiated and *Smed-H2B(RNAi) *samples**. Click here for file

Additional file 13 **List of 823 NB-expressed transcripts**. Click here for file

Additional file 14 **Summary of GO term annotation and enrichment analysis of NB-expressed transcripts**. Click here for file

Additional file 15 **Summary of domain annotation and enrichment analysis of NB-expressed transcripts**. Click here for file

Additional file 16 **Summary of KEGG pathway annotation and enrichment analysis of NB-expressed transcripts**. Click here for file

Additional file 17 **Compiled list of chromatin associated components**. Click here for file

Additional file 18 **Compiled list of putative CB components**. Click here for file

Additional file 19 **Compiled list of cell division machinery components**. Click here for file

Additional file 20 **Compiled list of transcription factors**. Click here for file

Additional file 21 **Compiled list of RNA associated transcripts**. Click here for file
