# Supplementary figures and images for: Defining the molecular profile of planarian pluripotent stem cells using a combinatorial RNA-seq, RNA interference and irradiation approach
Source: Genome Biol. 2012 Mar 22;13(3):R19. doi: 10.1186/gb-2012-13-3-r19 (PMC3439970; doi:10.1186/gb-2012-13-3-r19)

***Smed-nb.21.11e***

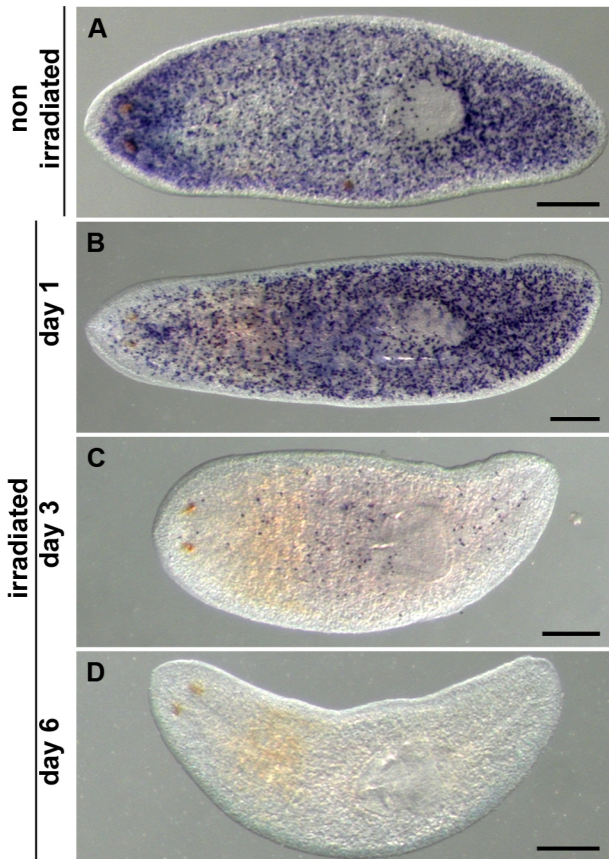

Supplement: Additional file 1 — Dynamics of Smed-nb.21.11e-positive cells after irradiation. (a-d) WMISH of Smed-nb.21.11e in non-irradiated (a) and irradiated animals 1 (b), 3 (c) and 6 (d) days after irradiation. Smed-nb.21.11e-positive cells are still detected 1 day after irradiation, but strongly decline in numbers after 3 days (b) and are not detectable after 6 days (d). Anterior is to the left. Scale bars: 500 μm. [file gb-2012-13-3-r19-S1.pdf]

# *Smed-agat-1*

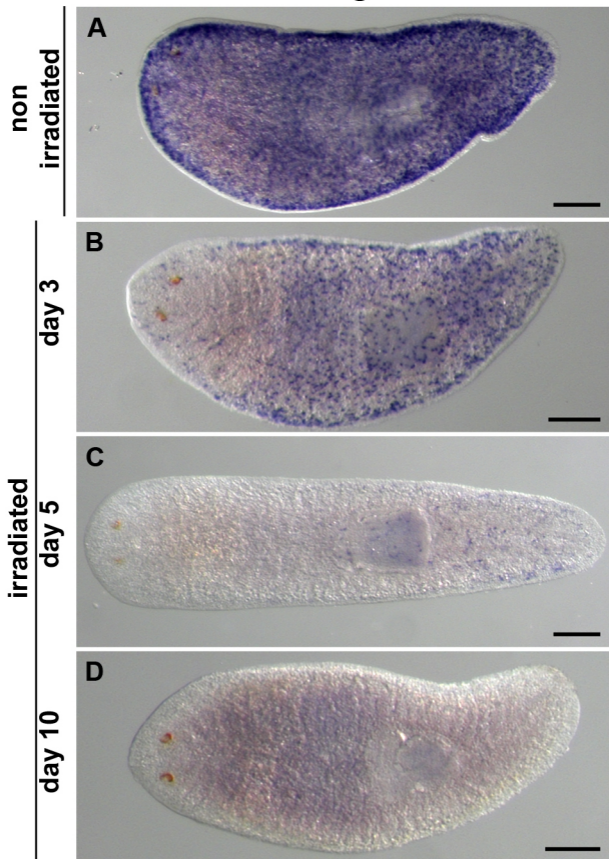

Supplement: Additional file 2 — Dynamics of Smed-agat-1-positive cells after irradiation. (a-d) WMISH of Smed-agat-1 in non-irradiated (a) and irradiated animals 3 (b), 5 (c) and 10 (d) days after irradiation. Smed-agat-1-positive cells are depleted from the anterior region of the worm 3 days after irradiation, strongly decline in numbers after 5 days (b) and are not detectable after 10 days (d). Anterior is to the left. Scale bars: 500 μm. [file gb-2012-13-3-r19-S2.pdf]

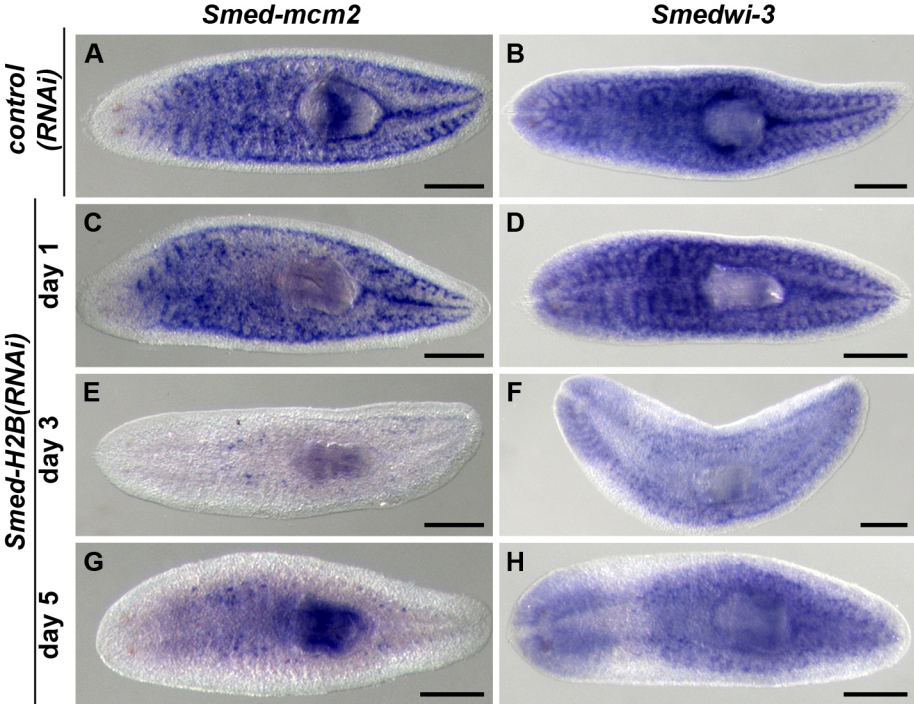

Supplement: Additional file 3 — Dynamics of expression of Smed-mcm2 and Smedwi-3 in Smed-H2B(RNAi) animals. (a-h) WMISH of Smed-mcm2 (a,c,e,g) and Smedwi-3 (b,d,f,h) in control(RNAi) (a,b) and Smed-H2B(RNAi) animals 1 (c,d), 3 (e,f) and 5 (g,h) days after RNAi. Most signals located in NBs disappear progressively for both markers (c-h). Almost no signals are detected 5 days after RNAi for the NB-specific marker Smed-mcm2 (g). The expression in the CNS of Smedwi-3 (h) is not eliminated by Smed-H2B RNAi and becomes more apparent after 5 days of RNAi (h). Some expression is detected in two rows of dorsal cells (g,h). Anterior is to the left. Scale bars: 500 μm. [file gb-2012-13-3-r19-S3.pdf]

# *Smed-nanos*

control  
(RNAi)

A

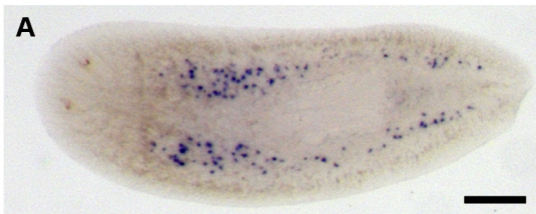

day 1

B

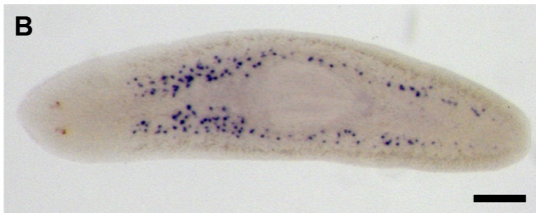

*Smed-H2B(RNAi)*

day 3

C

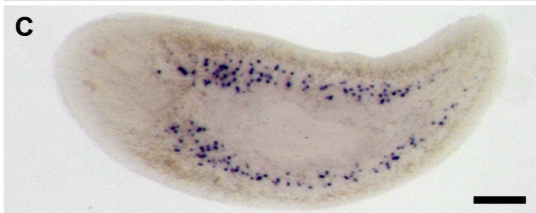

day 5

D

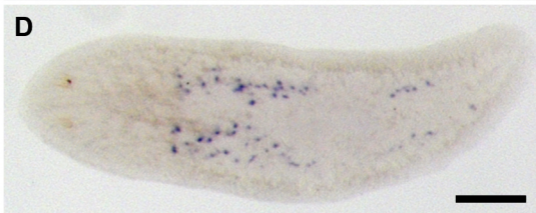

Supplement: Additional file 4 — Dynamics of Smed-nanos-positive cells after Smed-H2B RNAi. (a-d) WMISH of Smed-nanos in control(RNAi) (a) and Smed-H2B(RNAi) animals 1 (b), 3 (c) and 5 (d) days after RNAi. Smed-nanos-positive cells are distributed as two rows of NB-like dorsal cells, and are still detected, although severely reduced, 5 days after Smed-H2B RNAi (d). Anterior is to the left. Scale bars: 500 μm. [file gb-2012-13-3-r19-S4.pdf]

**A**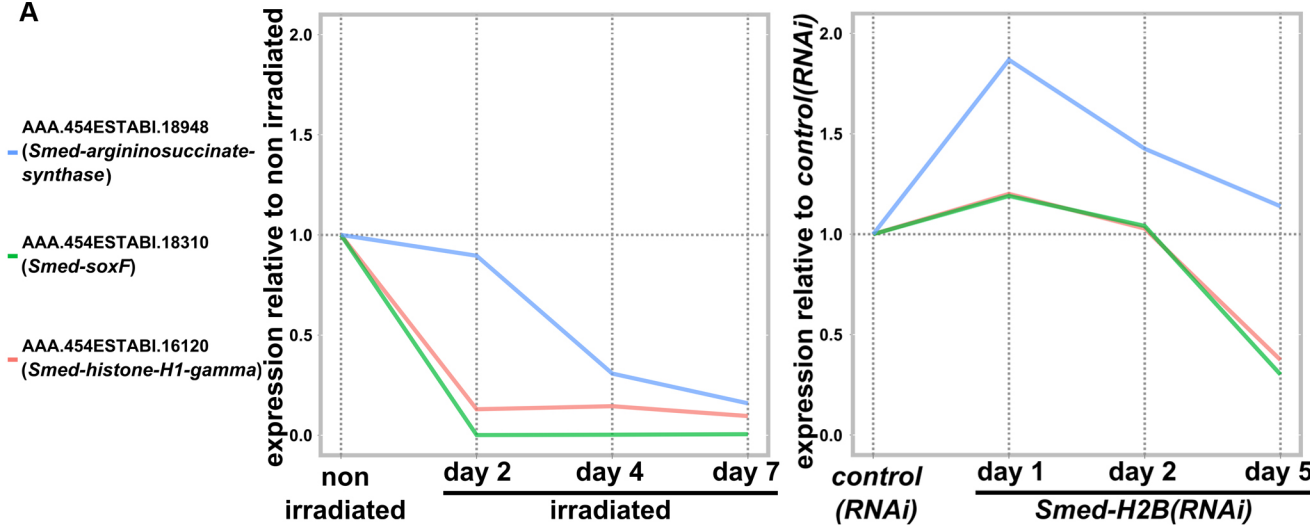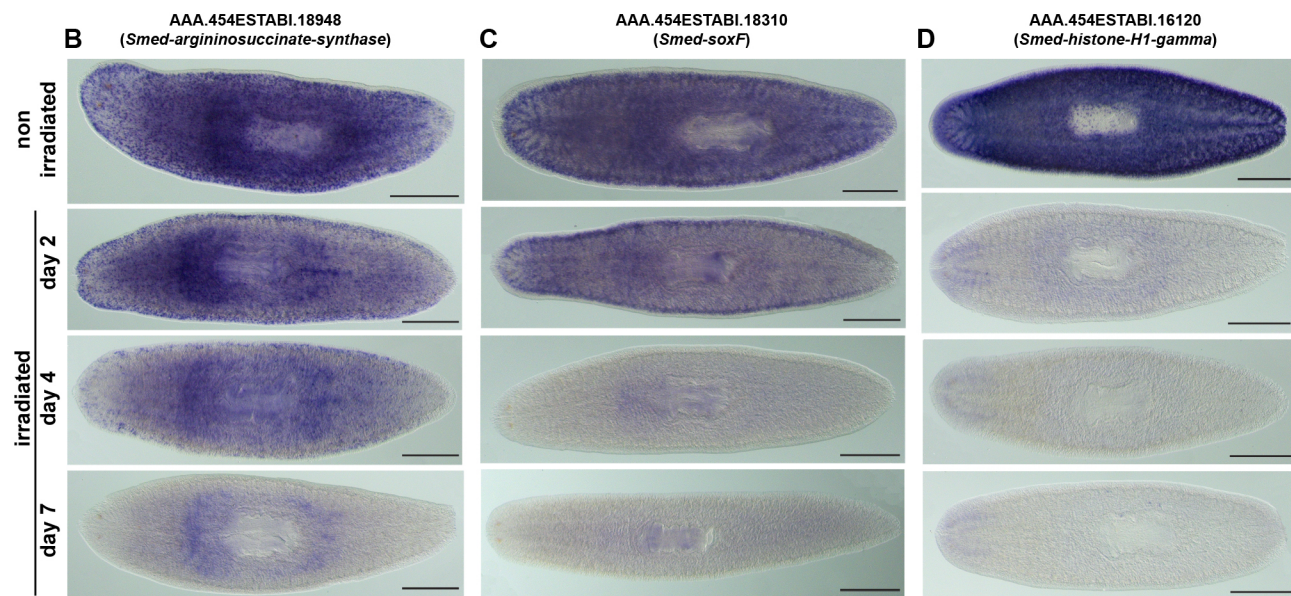

Supplement: Additional file 12 — Combined list of 1,270 transcripts downregulated in both irradiated and Smed-H2B(RNAi) samples. [file gb-2012-13-3-r19-S12.pdf]
